# Genetic Variability in *VEGFA* Gene Influences the Effectiveness of Tennis Elbow Therapy with PRP: A Two-Year Prospective Cohort Study

**DOI:** 10.3390/ijms242417292

**Published:** 2023-12-09

**Authors:** Paweł Niemiec, Alicja Jarosz, Anna Balcerzyk-Matić, Joanna Iwanicka, Tomasz Nowak, Tomasz Iwanicki, Marcin Gierek, Marcin Kalita, Wojciech Garczorz, Tomasz Francuz, Sylwia Górczyńska-Kosiorz, Wojciech Kania, Karol Szyluk

**Affiliations:** 1 Department of Biochemistry and Medical Genetics, School of Health Sciences in Katowice, the Medical University of Silesia in Katowice, Medyków 18 Str., 40-752 Katowice, Poland; pniemiec@sum.edu.pl (P.N.); abalcerzyk@sum.edu.pl (A.B.-M.); jiwanicka@sum.edu.pl (J.I.); tnowak@sum.edu.pl (T.N.); tiwanicki@sum.edu.pl (T.I.); 2 Center for Burns Treatment, Jana Pawła II Str., 41-100 Siemianowice Śląskie, Poland; marcin.gierek@clo.com.pl; 3 District Hospital of Orthopaedics and Trauma Surgery, Bytomska 62 Str., 41-940 Piekary Śląskie, Poland; marcin.kalita1991@gmail.com (M.K.); karol.szyluk@sum.edu.pl (K.S.); 4 Department of Biochemistry, School of Medicine in Katowice, Medical University of Silesia in Katowice, Medyków 18 Str., 40-752 Katowice, Poland; wojtekg@sum.edu.pl (W.G.); tfrancuz@sum.edu.pl (T.F.); 5 Department of Internal Medicine, Diabetology and Nephrology, School of Medicine with the Division of Dentistry in Zabrze, Medical University of Silesia in Katowice, 41-800 Zabrze, Poland; skosiorz@sum.edu.pl; 6 Department of Trauma and Orthopedic Surgery, Multidisciplinary Hospital in Jaworzno, Chełmońskiego 28 Str., 43-600 Jaworzno, Poland; wojtekkania@poczta.onet.pl; 7 Department of Physiotherapy, Faculty of Health Sciences in Katowice, Medical University of Silesia in Katowice, Medyków 12 Str., 40-752 Katowice, Poland

**Keywords:** tennis elbow, vascular endothelial growth factor, VEGFA, platelet-rich plasma, tendinopathy, genetic polymorphism

## Abstract

Vascular endothelial growth factor (VEGF) is implicated in both the etiology of tendinopathy and its healing process. Polymorphic variants of the *VEGFA* gene exhibit varied expression, which can influence the phenotype and treatment effectiveness. The aim of the present study was to analyze the influence of *VEGFA* gene variants on the effectiveness of tennis elbow therapy using platelet-rich plasma (PRP), measured through common patient-reported outcome measures (PROMs). A cohort of 107 patients (132 elbows) with tennis elbow was prospectively analyzed, with a two-year follow-up (at weeks 2, 4, 8, 12, 24, 52, and 104 after PRP injection). PROMs values were compared between variants of five *VEGFA* gene polymorphisms (rs699947 A>C, rs2010963 C>G, rs1413711 C>T, rs3024998 C>T and rs3025021 C>T) at each follow-up point. Patients with genotypes GG (rs2010963) and CC (rs3024998) had better response to PRP therapy (significantly fewer symptoms and limitations in the upper limb compared to carriers of alleles C and T, respectively). Polymorphisms influenced also selected hematological parameters. *VEGFA* gene polymorphisms (rs2010963 and rs3024998) appear to be significant treatment modifiers for tendinopathy, and their genotyping may serve as an effective tool for personalized patient selection for PRP therapy.

## 1. Introduction

Tennis elbow (TE) is an overload tendinopathy of the extensor carpi radialis brevis (ECRB) tendon at the tissue level characterized by an increase in tenocyte proliferation, disorganization of the extracellular matrix, an increase in microcirculation and innervation of sensory nerves, and an increased number of inflammatory cells [1,2,3]. The final stage of tendinopathy is characterized by degenerative features, including abnormal tendon structure and neovascularization. Many types of cytokines may play a role in the etiology of TE, including vascular endothelial growth factor (VEGF).

**Figure 1:**
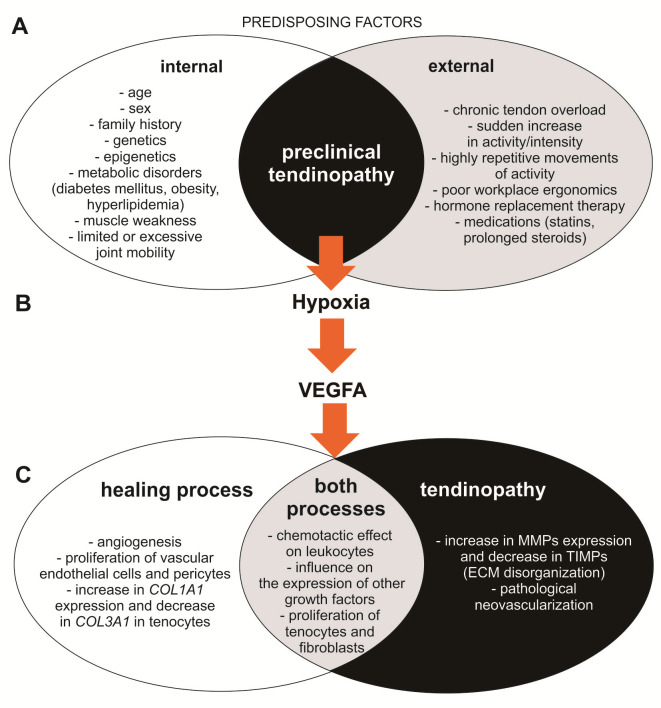
Involvement of VEGF in the physiology and pathology of tendons after injury. Based on [1,2,3,4,5,6,7,8,9,10,11]. Tendinopathy is a multifactorial condition (**A**). Its subclinical form usually arises from chronic or acute tendon overload, while overlapping predisposing factors (biological, coexisting diseases, or certain medications) can exacerbate the patient’s condition. Damage to tendon structures leads to hypoxia (**B**), during which there is an increased secretion of hypoxia-inducible factor-1 (HIF-1). HIF-1, mechanical tendon overload, neuronal signals, and some pro-inflammatory cytokines are the main factors that increase *VEGFA* expression. VEGFA can promote events leading to both healing and the manifestation of the clinical form of tendinopathy (**C**). In the early stages of the tendon injury response, VEGFA participates in the restoration of microcirculation by stimulating endothelial cell division. It also intensifies the proliferation of pericytes, tenocytes, and fibroblasts. Angiogenesis also promotes the chemotaxis of monocytes and granulocytes and increases the availability of other growth factors. Both of these processes, along with increased proliferation of tenocytes and fibroblasts, are observed during both tendon healing and tendinopathy. During tendinopathy, a remodeling of the extracellular matrix (ECM) is also observed, involving the loss of type 1 collagen and its replacement with type 3 collagen. VEGFA promotes these processes by influencing the expression of matrix metalloproteinases (MMP) and inhibiting the expression of tissue inhibitors of metalloproteinases (TIMP) in endothelial cells and fibroblasts, leading to the destruction of type 1 collagen. On the other hand, VEGFA also participates in restoring the initial proportions of collagen during the healing process, stimulating the expression of *COL1A1* (encoding type 1 collagen chains) in tenocytes and reducing the expression of *COL3A1* (encoding type 3 collagen chains). The pathological neovascularization observed in tendinopathy is the result of the prolonged influence of VEGFA on the damaged tendon, disrupting its biomechanical properties.

The VEGF family consists of several members [4,5,6], among which the most attention is focused on VEGFA, due to its key role in the regulation of angiogenesis [6]. In the case of VEGFA, signaling through the vascular endothelial growth factor receptor 2 (VEGFR2) promotes angiogenesis, whereas VEGFR1 inhibits angiogenesis as a “decoy receptor” [7]. Angiogenesis occurs through a variety of physiological processes, including wound healing, however, pathological neovascularization is one of the main features of tendinopathy. The involvement of VEGFA in the pathogenesis of tendinopathy as well as in the tendon healing process seems indisputable, but its role is not unidirectional and obvious, and probably depends on the stage of the disease and overall clinical condition. It has been shown that the presence of VEGFA and angiogenesis influences tendon healing at the early stage of tendon repair, but the persistent high VEGF expression may impair tendon repair at a later stage [5]. According to the literature review by Vasta et al. [8], neovascularization observed in tendinopathy is primarily driven by VEGF. In normal, asymptomatic tendons, the expression of VEGF is largely suppressed, whereas in tendons subjected to chronic or cyclic overload, the expression of VEGF is significantly increased in both the early and late phases of the overload process [8]. On the other hand, VEGFA influences skeletal development and bone repair. It may regulate the differentiation of skeletal stem cells from bone marrow, periosteum, and surrounding muscles into either chondrocytes or osteoblasts [9]. VEGFA also enhances proliferation of human tenocytes, pericytes and fibroblasts [10] and leads to up-regulation of the *COL1A1* gene and down-regulation of the *COL3A1* gene in human tenocytes [5]. During tendon damage, type III collagen is first synthesized and in physiological conditions is replaced by type I collagen. However, one of the main features of tendinopathy is the disorganization of the extracellular matrix due to a change in the ratio of type I to type III collagen. An increased ratio of type III collagen to type I collagen is observed, and the persistence of tendinopathy symptoms results from a delay in restoring their normal ratio [1,2,11]. The involvement of VEGFA in the physiology and pathology of the tendon after injury is shown in Figure 1.

VEGFA is encoded by the *VEGFA* gene (6p21.1), which is highly polymorphic. It has been shown that certain polymorphisms of the *VEGFA* gene influence variable gene expression and are associated with mRNA and/or protein levels [12,13,14]. To date, they have been studied in relation to the occurrence of neoplastic diseases [15,16], cardiovascular conditions [17,18], and metabolic disorders [19]. There are also limited data suggesting that *VEGFA* gene polymorphisms may be involved in tendon-related disorders [20,21]. For these reasons, we conducted our own research with the primary aim of investigating whether selected *VEGFA* gene polymorphisms influence the effectiveness of tennis elbow treatment, as assessed by the results of common patient-reported outcome measures (PROMs) such as VAS (visual analog scale), QDAH (quick version of disabilities of the arm, shoulder and hand score), and PRTEE (patient-rated tennis elbow evaluation), during a two-year observation.

The current research is a continuation of a larger study on the influence of genetic factors on the effectiveness of platelet-rich plasma (PRP) therapy in the treatment of tennis elbow. So far, we have analyzed genes encoding platelet-derived growth factor (PDGF) subunits (*PDGFA* and *PDGFB* genes) and PDGF beta receptor (*PDGFRB* gene) [22,23,24].

## 2. Results

### 2.1. General Characteristics

The study group contained 107 patients (132 elbows), including 25 bilateral patients. There were 65 females and 42 males, aged 24–64 years (median ± QD: 46.00 ± 5.50). Hypertension, thyroid disease, and gout were the most common comorbidities. The median concentration of white blood cells (WBC) was 6.26 ± 1.16 (10^9^/L ± QD). The median platelet (PLT) level in the whole blood was 240.00 ± 40.50 (10^9^/L ± QD). Females had a higher concentration of platelets than males (261.50 ± 33.00 vs. 224.00 ± 38.75, respectively, *p* = 0.000) as well as higher value of plateletcrit (2.37 ± 0.36 vs. 2.04 ± 0.33, respectively, *p* = 0.001). The mean platelet volume (MPV) in the whole blood was 9.10 ± 0.73 (fL ± QD), and there was no statistical difference between the sexes (*p* = 0.125). Platelet parameters of PRP also did not differ between sexes. Table 1 summarizes basic demographic and clinical parameters.

### 2.2. Genetic Characteristics

Genotyping was successful in all patients. Data on genotype and allele frequencies are presented in Table 2. The genotype distribution of individual SNPs was consistent with the Hardy–Weinberg equilibrium. Only in the case of the rs1413711 polymorphism were the observed genotype frequencies differed significantly from the predicted ones (Table 2).

**Figure 2:**
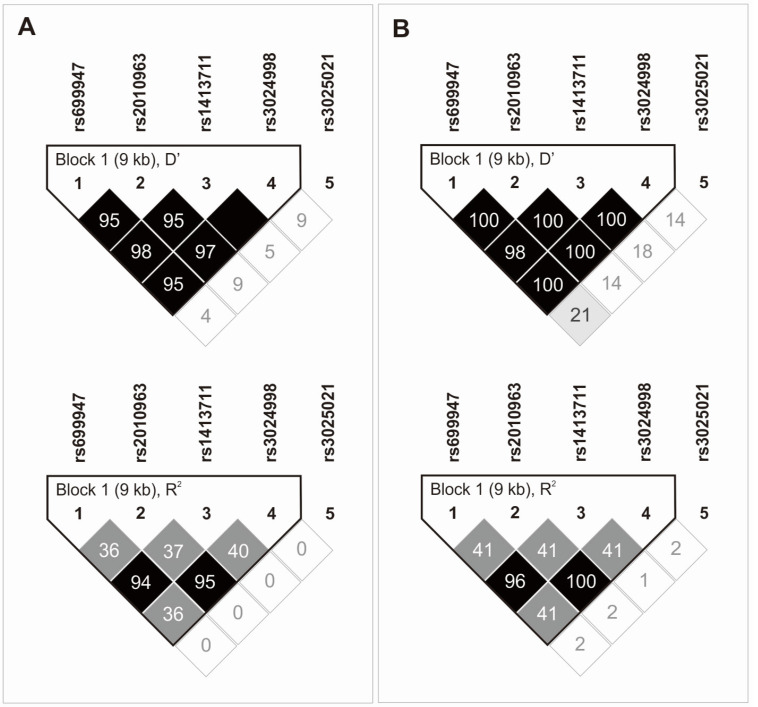
Haplotype analysis of *VEGFA* gene polymorphisms in the study group (**A**) and CEU (U.S. Utah residents with ancestry from northern and western Europe) population (**B**).

Haplotype analysis revealed the presence of a 9 kB haplotype block created by the first four polymorphisms (in the order consistent with their location on chromosome 6). The last one, namely rs3025021, was not in linkage disequilibrium (LD) with any of these four SNPs (Figure 2A). Almost identical dependencies also apply to the CEU (U.S. Utah residents with ancestry from northern and western Europe), population (Figure 2B).

The AT (rs699947 and rs1413711, respectively) and GC (rs2010963 and rs3024998, respectively) alleles were in the strongest LD. The second diplotype was also characterized by the highest frequency in the study group (0.715%). Detailed data on the frequency of haplotypes and diplotypes of analyzed polymorphisms are presented in Table 3.

### 2.3. VEGFA Gene Polymorphisms and the Effectiveness of PRP Therapy

The effectiveness of PRP therapy in relation to VEGFA gene variants was analyzed both in the additive model, comparing PROMs values between individual genotypes, and in the recessive/dominant model, comparing PROMs values between homozygotes of a given type and carriers of the second allele.

In the additive model, only rs2010963 and rs3024998 polymorphisms influenced the effectiveness of PRP therapy (Table 4). GG (rs2010963) homozygotes achieved better treatment effects at weeks 12 (QDASH and ΔQDASH) and 104 (QDASH, ΔQDASH and PRTEE) than CG heterozygotes. Differences in PROMs values between the CC and CG genotypes, as well as CC and GG, did not show statistical significance. In the case of the rs3024998 polymorphism, showing strong LD with rs2010963, the genotype associated with better therapy effectiveness was CC (Table 4). Detailed data for all polymorphisms are presented in Table S1.

**Figure 3:**
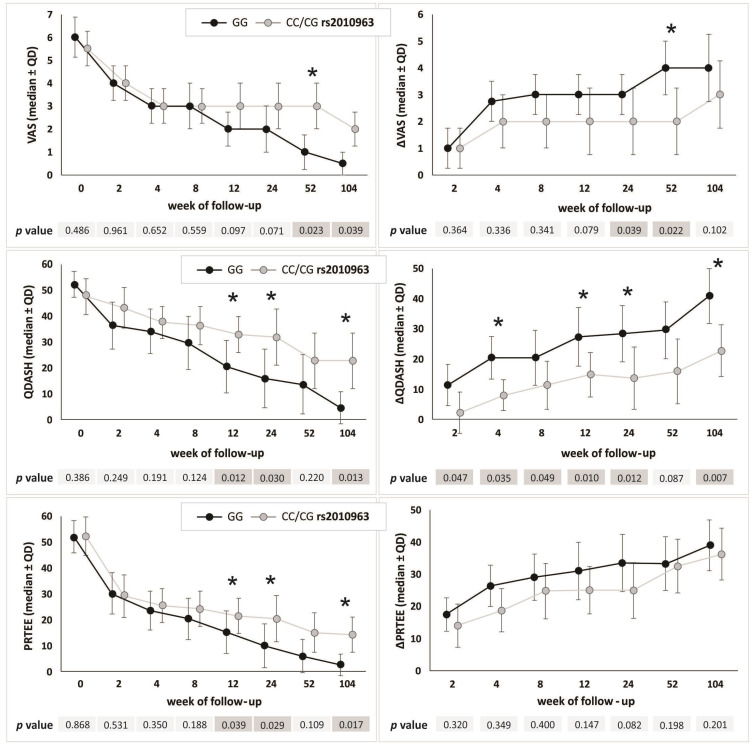
Medians (±QD) of PROMs values in respect to genotype variants of the *VEGFA* gene rs2010963 polymorphism (recessive/dominant model). Legend: QD, quartile deviation; PROM, patient-reported outcome measure; VAS, visual analog scale; QDASH, quick version of disabilities of the arm, shoulder and hand score; PRTEE, patient-rated tennis elbow evaluation; *, differences remaining significant (*p* < 0.050) after removing diabetics from the analysis.

In the recessive/dominant model, only rs2010963 and rs3024998 polymorphisms also differentiated the response to PRP therapy (detailed data for each polymorphism are provided in Tables S2–S6). GG (rs2010963) homozygotes were characterized by a better response to therapy due to significantly lower VAS values (weeks 52 and 104 of follow-up), significantly higher ΔVAS values (weeks 24 and 52), significantly lower QDASH values (weeks 12 and 24 and 104), higher ΔQDASH values (weeks 2–24 and 104) and lower PRTEE values (weeks 12, 24 and 104) than carriers of the C allele (Figure 3, Table S3).

The results for the rs3024998 polymorphism were similar, with statistically significant differences between CC homozygotes (better response to PRP therapy) and carriers of the T allele (worse PROMs parameters) regarding: VAS (week 52 of follow-up), ΔVAS (weeks 24 and 52), QDASH and ΔQDASH (weeks 12, 24 and 104), and PRTEE (weeks 24 and 104). Detailed data are presented in Table S5.

### 2.4. VEGFA Gene Polymorphisms and Whole Blood and PRP Parameters

**Figure 4:**
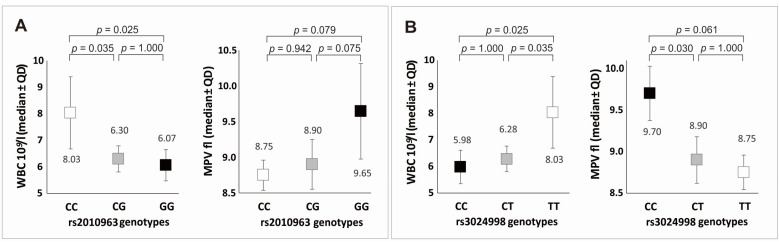
White blood cells (WBC) and mean platelet volume (MPV) values in individuals with particular genotypes of *VEGFA* gene polymorphisms (additive model): (**A**) for rs2010963; (**B**) for rs3024998.

It was checked whether there are differences in the values of whole blood and PRP parameters between variants of individual polymorphisms. The additive model showed that patients with genotypes associated with a better response to PRP therapy are characterized by the lowest WBC concentrations and the highest MPV values in whole blood (Figure 4), and in the latter case, statistically significant differences concerned only rs3024998 polymorphism (*p* = 0.018 in Kruskal–Wallis test, *p* > 0.050 in post hoc analyses for rs2010963 genotypes). Platelet parameters in PRP did not differentiate the genotypes of individual SNPs in the additive model (Table S7).

In the recessive/dominant model, it was shown that GG homozygotes (rs2010963) were characterized by higher MPV and EOS% levels in whole blood than C allele carriers, while CC homozygotes (rs3024998) had lower platelet concentrations, higher MPV and EOS% levels in whole blood, and higher levels of MPV and PDW in PRP compared to T allele carriers (Table 5). Detailed data for all analyzed *VEGFA* gene polymorphisms are summarized in Table S8.

Analysis of the VEGF protein concentration in the PRP preparation was performed with the VEGF Quantikine ELISA Kit. The sensitivity of the test was 9 ng/mL. VEGF concentration was detectable in approximately 15% of samples. In most subjects, it did not exceed the detection threshold.

### 2.5. VEGFA Gene Polymorphisms and Clinical Phenotype

There were no statistically significant differences in the median age, BMI, number of cigarettes smoked per day, or differences in the frequency of using additional forms of treatment for tennis elbow (physiotherapy, NSAIDs) during follow-up between patients with particular genotypes of the tested polymorphisms (Table S9).

Homozygotes GG (rs2010963) and CC (rs3024998) responding better to PRP therapy were characterized by significantly higher alcohol consumption per week than patients with other genotypes (Kruskal–Wallis test: *p* < 0.010 in both cases) (Table S9). This effect also occurred in the recessive/dominant model (Table 6). Homozygotes GG (rs2010963) and CC (rs3024998) also had a higher frequency of cigarette smokers and a lower frequency of diabetes mellitus than carriers of the C (rs2010963) and T (rs3024998) allele (Table 6). In the case of diabetes mellitus, statistically significant differences in its frequency were found between selected genotypes of both polymorphisms only in the additive model (Table S9). Removing patients with diabetes mellitus (n = 4) from the analysis did not have a significant impact on the results regarding the effectiveness of therapy (Section 2.3) in the additive model (Table 4). Small differences, while maintaining the trend, concerned the results of recessive/dominant model (Figure 3, Tables S3 and S5).

## 3. Discussion

In the present study, we investigated whether *VEGF* gene polymorphisms influence the effectiveness of tennis elbow therapy, as measured via commonly used patient-reported outcome measures such as VAS, QDASH, and PRTEE. The results obtained indicate that the best response to PRP therapy was characterized by individuals who were homozygous for GG and CC genotypes of two polymorphisms, specifically rs2010963 and rs3024998, which were in strong linkage disequilibrium. The observed effect was independent of age, gender, the use of additional forms of tennis elbow therapy during follow-up, and most blood parameters. However, the homozygous individuals showing a better response to therapy had higher MPV values and lower WBC concentration in whole blood, consumed more alcohol per week, and exhibited significantly higher nicotine use rates.

VEGFA regulates the proliferation and migration of endothelial cells, which, in turn, leads to the formation of new vascular structures. For these reasons, increased expression of the gene encoding VEGFA can lead to microvascular pathologies. Unfortunately, there is currently no literature data regarding the impact of the rs3024998 polymorphism on the expression of the *VEGFA* gene. However, previous studies indicate the role of carrying the T allele of the rs3024998 polymorphism in the *VEGFA* gene in shaping the risk of microvascular complications, including retinopathy. This particular variant may induce increased blood vessel permeability, characterized by excessive perfusion [25]. While this relationship has been demonstrated in diabetic patients, VEGFA also stimulates angiogenesis in tendinopathy, where immature vessels may be responsible for persistent hypoxia in neovascularization areas [26]. In such conditions, hyperpermeable new vessels do not provide an adequate supply of oxygen and nutrients necessary to maintain tissues and their potential regeneration [27]. There is a suspicion that these processes may contribute to poorer prognoses and difficulties in achieving therapeutic effects in a subgroup of our patients who carry the T allele of the rs3024998 polymorphism.

While there are no data regarding the association of the rs3024998 polymorphism with serum VEGFA levels, it should be noted that the studied variant is in strong linkage disequilibrium with another analyzed polymorphism, namely rs2010963. The rs2010963 polymorphism is located in the regulatory region of the 5′UTR gene and affects gene expression at both the transcriptional and translational levels. Studies by Chen et al. confirm the association of the C allele with higher transcriptional activity [28]. In our current study, GG homozygotes (rs2010963) exhibited a better response to PRP therapy than carriers of the C allele. In another study [29], in a group of patients with recurrent glioblastomas, higher levels of VEGFA were observed in the CC homozygote group. This genotype was also associated with an increased risk of thrombotic and hemorrhagic events, which could be a consequence of promoting VEGFA-dependent angiogenesis. Considering the linkage of the rs3024998 and rs2010963 polymorphisms, it can be hypothesized that the reduced effectiveness of PRP therapy in carriers of the T allele (rs3024998) and the C allele (rs2010963) may have a common denominator in the form of increased expression of the *VEGFA* gene and intensified angiogenesis in injury areas [29].

Understanding the gene expression of *VEGFA* in the context of the rs2010963 polymorphism is complicated by the fact that different results have been obtained in some studies. In patients with diabetic foot ulcers, the VEGFA concentration was reduced in the case of the CC genotype [30]. Watson et al. [31] observed a similar relationship and suggested that this polymorphism is located at the binding site of the transcription factor MZF1 (myeloid zinc finger 1), affecting its binding specificity and, consequently, the VEGFA level. There are also studies that have not shown a relationship between the rs2010963 polymorphism and VEGFA levels. In a study by Sudhesan et al. [32], involving 300 patients with psoriasis and joint inflammation as well as 300 healthy individuals, the serum VEGFA level did not significantly differ between the three genotypes either in the patient group or the control group. However, statistically significant differences in serum VEGFA levels were observed between patients and the control group with a particular genotype [32]. It is highly likely that the influence of the rs2010963 polymorphism on protein levels may be modulated by the patient’s clinical condition, including the presence of comorbidities, as suggested by inconsistencies observed in the literature. Unfortunately, we cannot refer to literature data on this issue because the sensitivity range of the ELISA test used to assess the VEGF protein level in the present study did not allow for the determination of VEGF concentration, including in the context of *VEGFA* gene polymorphisms. The protein concentration in PRP was below the detection threshold for most of the patients.

From the literature review, it also appears that the rs2010963 polymorphism has been associated with musculoskeletal diseases, including tendon disorders. In the population of Polish Caucasians, a link was established between the rs2010963 polymorphism and the risk of anterior cruciate ligament rupture (ACLR), with the carrier state of the C allele and CC homozygosity being identified as ACLR risk factors [20]. It is worth noting that the risk variants from this study are the same as those in our study that were associated with poorer PRP therapy effectiveness. In another work, three *VEGFA* gene polymorphisms were analyzed, including two of those examined in the present study, namely rs699947, rs1570360, and rs2010963. It was found that the A-G-G haplotype was associated with an increased risk of Achilles tendon inflammation [21].

The significance of the *VEGFA* gene polymorphisms we studied in the context of the observed differences in blood platelet levels, mean platelet volume (MPV), and EOS% among patients with varying treatment effectiveness remains unclear. Literature data suggest that VEGFA may indirectly participate in hematopoiesis through intracellular autocrine signaling that controls the homeostasis of hematopoietic stem cells [33]. Additionally, considering specific morphological elements of blood, it should be noted that eosinophils play an important role in tissue regeneration, and their damage promotes their rapid recruitment to inflammatory sites [34]. They also release platelet-activating factors, which can facilitate tissue regeneration in patients exhibiting better treatment outcomes [35]. However, given the complexity of the mechanisms underlying individual responses to PRP therapy, further research is needed to fully understand these processes.

In the current analysis, we did not detect factors that could significantly influence the results regarding the impact of *VEGFA* gene polymorphisms on PRP therapy effectiveness. The frequency/median distributions of demographic and clinical factors were similar among patients with different genotypes. An exception was the frequency of diabetes, which was higher in carriers with poorer PRP therapy efficacy. Diabetes is considered a risk factor and a cause of chronic tendinopathy with unfavorable therapeutic outcomes [36,37]. However, in this study, it was demonstrated that differences in diabetes frequency between genotype variants did not show statistical significance in the recessive/dominant model, and the exclusion of diabetic patients from the statistical analysis did not affect the nature and direction of the observed relationships in both models. As for other factors, it appears surprising to the authors that there was a higher frequency of cigarette smokers and higher alcohol consumption among homozygotes for whom PRP therapy was more effective. The higher alcohol consumption among homozygotes with the CC genotype (rs3024998) may partially explain why these homozygotes had a lower platelet concentration in whole blood than carriers of the T allele. During the study, a weak negative correlation was observed between PLT concentration and the amount of alcohol units consumed during the week (r_s_ = −0.31, *p* < 0.002).

The current study has several limitations. One of them is the relatively small study group. However, in our favor, the analyses mainly focused on quantitative data (PROMs), which are less sensitive to statistical analysis than qualitative data. Another positive aspect is the clinical and ethnic homogeneity of the group, which is crucial in the analysis of associations. Another limitation of the current research is the inclusion of patients who, during the follow-up period after PRP injection, used additional forms of therapy (NSAIDs, physiotherapy techniques). However, in the comparative analysis, we found that the frequency of their use did not significantly differ among patients with different *VEGFA* gene variants, suggesting that they did not influence the observed results. Additionally, prohibiting patients from using other forms of therapy, such as pain relief, seems deeply unethical to us. No control group, untreated with PRP, is also a limitation of the current study. Conducting research in a treated group, for example, using only physiotherapeutic methods, would allow for a comparative analysis of the impact of *VEGFA* genotypes on the effectiveness of tendinopathy treatment in patients who were and were not provided with VEGF through injection.

In conclusion, the current study indicates that the GG (rs2010963) and CC (rs3024998) genotypes, which are in strong linkage disequilibrium in the Caucasian population, are associated with a better response to PRP therapy in the treatment of tennis elbow. Patients with these variants report fewer symptoms and limitations in the upper limb than carriers of the C and T alleles, respectively. This study complements our knowledge about factors contributing to the effectiveness of treating tendinopathy with PRP. Genotyping one of these two polymorphisms may become an effective tool for patient selection for PRP therapy in the future. However, considering the multifactorial aspect of both the etiology of tendinopathy and the healing process, further basic and clinical research is needed to expand our understanding of the role of *VEGFA* polymorphisms in these processes.

## 4. Materials and Methods

### 4.1. Study Design

This prospective cohort study was performed in accordance with STROBE and MIBO guidelines. The patients were followed up for two years (at 2, 4, 8, 12, 24, 52, and 104 weeks after injection), and common patient-reported outcome measures (VAS, QDASH and PRTEE) were recorded. Five single-nucleotide polymorphisms of the *VEGFA* gene were genotyped, and the effectiveness of PRP therapy was compared between respective genotypic variants in additive and recessive/dominant models. An analysis of polymorphisms was also carried out in the context of the clinical phenotype, comorbidities and parameters of whole blood and PRP preparation.

### 4.2. Patients

This patient group was presented in detail in our previous studies [22,23,24]. It was a cohort of 107 patients (132 elbows, 100%) with lateral elbow tendinopathy (M77.1., according to the International Statistical Classification of Diseases and Related Health Problems 10th Revision, ICD-10) treated with autologous PRP. Patients were in the study between November 2018 and November 2019, and they were selected for the study, examined, and injected by the same orthopedic surgeons (K.S. and W.K.), following the same study protocol. Follow up data was collected until November 2021.

**Figure 5:**
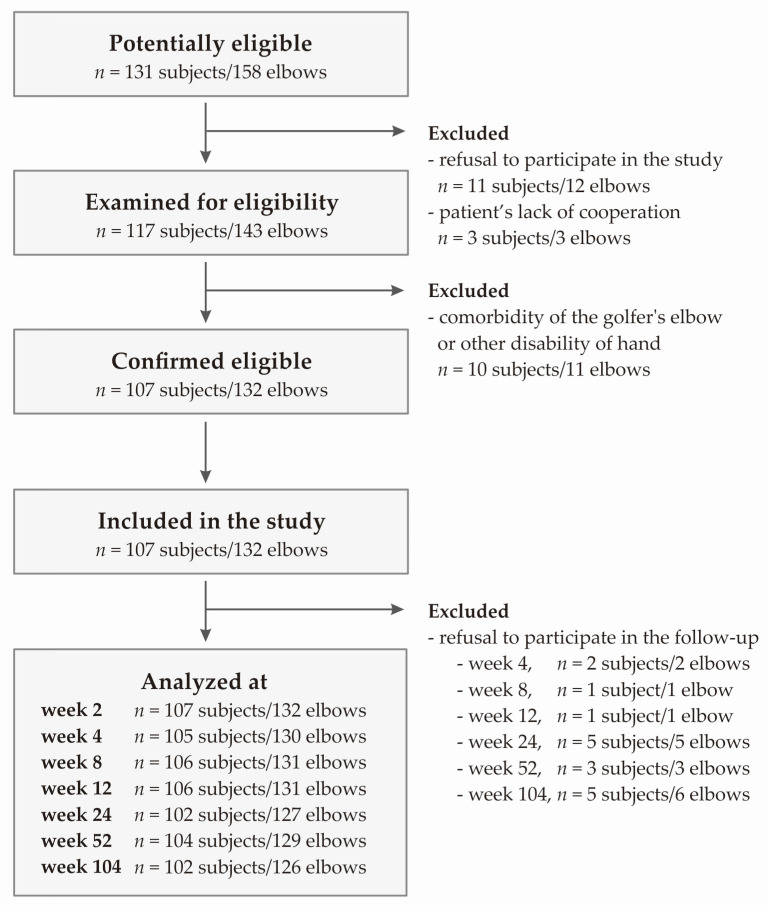
Flowchart of the study selection.

The study included patients with typical TE symptoms lasting at least three months before injection, such as pain in the region of common extensor origin radiating distally and proximally, muscle pain and weakness, morning stiffness, positive Thomson’s and Mill’s tests, and Cozen’s sign as well as tenderness during palpation of the lateral epicondyle of the humerus. The exclusion criteria were additional injury/disease of affected limb, prior surgical intervention, rheumatoid arthritis, pregnancy, active malignancy, cervical radiculopathy, current anti-platelet medication, local steroid injections in the preceding 6 months, previous PRP injections and cognitive limitations. The flow chart of patient selection is presented in Figure 5.

There was no formal post-injection rehabilitation protocol. Further post injection therapy (physiotherapy, nonsteroidal anti-inflammatory drugs, steroids) was monitored during the follow-up period but was not a criterion for exclusion.

### 4.3. PRP Separation and Injection Procedure

Blood collection, separation and injection of PRP were performed in standardized conditions (20 °C, same light exposure). Since all procedures have been described in our previous studies [22,23,24], they will be briefly presented here. PRP was separated immediately after blood collection using manufacturer’s instructions (Autologous Conditioned Plasma, Arthrex GmbH, Munich, Germany). 12 mL of whole blood was mixed with 3.13% sodium citrate (MediPac GmbH, Königswinter, Germany) in a ratio of 9:1 and then centrifuged for 5 min using a Rotofix 32A centrifuge (Andreas Hettich GmbH & Co., Tuttlingen, Germany) at a speed of 1500 rpm.

During separation, between 2.5 and 3.5 mL of PRP were obtained and a volume of 2.0–3.0 mL was injected immediately after centrifugation in the common extensor origin area. The injection was performed under ultrasound guidance using Mindray DC-3 apparatus (Mindray North America, Mahwah, NJ, USA) with a linear probe (frequency range of 5, 7.5, 10 MHz). The remaining 0.5 mL of PRP was saved for further analysis. The injections were performed by two senior trauma and orthopedic consultants (K.S. and W.K.) with 17 years of experience.

After the procedure, patients were advised to avoid heavy use of the affected limb for 24 h. No infection at the injection site was observed in any of the patients.

### 4.4. Whole Blood and PRP Parameters

On the day of PRP injection, a complete blood count in fresh blood was performed, as was as an analysis of platelet parameters (platelet concentration, PLT; plateletcrit, PCT; mean platelet volume, MPV; and platelet distribution width, PDW) in the PRP. For patients with bilateral tennis elbow, if the injections were carried out on different dates, separate analyses (whole blood and PRP) were performed. The remaining volume of PRP was immediately frozen and stored at −86 °C. The concentration of VEGF in PRP was assessed using the VEGF Quantikine ELISA Kit (R&D Systems Inc., Minneapolis, MN, USA) in undiluted material according to the manufacturer’s instructions.

### 4.5. Follow-Up, Outcomes, Measures of Effectiveness

The effectiveness of PRP therapy was analyzed and compared to clinical condition (pain and disability of affected limb) on the day of injection (baseline, week 0). Follow-up review was performed at 2, 4, 8, 12, 24, 52, and 104 weeks.

The VAS, QDASH, and PRTEE questionnaires were used for assessment of pain and disability, with the following ranges assumed: from 0 minimum to 10 maximum pain for VAS and from 0 minimum to 100 maximum pain and disability for QDASH and PRTEE. At each follow-up point, raw outcome values (VAS, QDASH, and PRTEE) and differences from baseline (ΔVAS, ΔQDASH, and ΔPRTEE) were used to determine the effectiveness of therapy in relation to individual variables, including *VEGFA* gene variants.

### 4.6. Genetic Analyses

Genomic DNA was isolated from peripheral blood leukocytes using the MasterPure genomic DNA purification kit (Epicenter Technologies, Madison, WI, USA). SNPs of the *VEGFA* gene were genotyped using the TaqMan Predesigned SNP Genotyping Assay kits and the 7300 Real-Time PCR System (Thermo Fisher Scientific, Pleasanton, CA, USA). The accuracy of genotyping was checked by re-genotyping 10–15% of samples. The repeatability of results was 100%.

**Figure 6:**
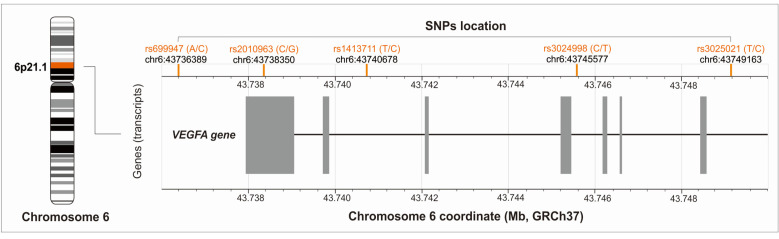
Location of the studied polymorphisms on chromosome 6 (the figure was created on the basis of data from LDmatrix Tool [39]).

Only SNPs with minor allele frequency ≥ 20% in populations of European origin (CEU, U.S. Utah residents with ancestry from northern and western Europe) based on the Database of SNPs of National Center for Biotechnology Information, U.S. National Library of Medicine [38] were selected. There were rs699947 (A>C), rs2010963 (C>G), rs1413711 (C>T), rs3024998 (C>T), and rs3025021 (C>T) variants. The location of the analyzed SNPs on chromosome 6 is shown in Figure 6.

### 4.7. Statistical Analyses

Data were analyzed using the Statistica 13.0 software (TIBCO Software Inc., Palo Alto, CA, USA). Normality of distribution of quantitative data was assessed using the Shapiro–Wilk test. Since all analyzed quantitative variables had a non-normal distribution, they were reported as medians and their spread as quartile deviation (QD). Non-parametric tests were used for comparisons, such as the Mann–Whitney U test (in the case of dichotomous grouping variables) and the Kruskal–Wallis test (in the case of grouping variables with more than two categories). Spearman’s rank correlation coefficient (r_s_) was used as a measure of the correlation between quantitative variables. Cases with missing data were rejected from the respect comparisons.

Genetic data were analyzed in additive and dominant/recessive models of inheritance. The Hardy–Weinberg equilibrium was tested using a χ^2^ test as well as comparisons of genotype variants frequencies between categories of qualitative variables. Yates correction was used for subgroups with less than ten subjects. Haplotype blocks in the study group were calculated using the HaploView 4.2 software [40] using the Gabriel et al. algorithm [41]. Haplotype blocks of CEU population were defined with the use of LDmatrix Tool [39]. The values of D′ and R^2^ were used as linkage disequilibrium measures. Study size and power analysis were computed using Sample size t test—median and SD tool [42]. The power of all statistically significant tests in the current work was greater than 85%, with a 95% two-sided confidence level. Statistical significance was accepted at *p* < 0.050. In cases of multiple comparisons, the *p* values were adjusted using the Bonferroni correction.

## 5. Conclusions

In summary, our two-year study emphasizes the significant influence of specific *VEGFA* gene polymorphisms, namely rs2010963 and rs3024998, on the effectiveness of tennis elbow treatment. Patients with GG (rs2010963) and CC (rs3024998) genotypes exhibited better responses in patient-reported outcome measures, suggesting a potential role for these genetic markers in predicting treatment success. Furthermore, the association between *VEGFA* gene polymorphisms and hematological parameters highlights the complexity of tendinopathy and its treatment. These findings underscore the importance of considering genetic factors in tailoring tennis elbow treatment strategies, offering potential opportunities for more personalized and effective interventions. Further research on larger and diverse populations are necessary to better understand *VEGFA* gene polymorphism-dependent mechanisms, which may help optimize the diagnosis and treatment of tendinopathy in the future.

## Figures and Tables

**Table 1:** Demographic and clinical characteristics of the study group (baseline week 0).

Characteristics			
General	number of subjects, N	107	-
	number of elbows, n (%)	132	(100.0)
	tennis elbow in the dominant hand, n (%)	86	(65.2)
	age, median ± QD	46.00	5.50
	BMI, median ± QD	25.65	2.00
	current smokers, n (%)	22	(16.6)
Comorbidities	diabetes mellitus, n (%)	4	(3.0)
	gout, n (%)	8	(6.1)
	thyroid diseases, n (%)	15	(11.4)
	hypertension, n (%)	18	(13.6)
Whole Blood	PLT 10^9^/L, median ± QD	240.00	40.50
parameters	PCT ml/L, median ± QD	2.31	0.36
	MPV fL, median ± QD	9.10	0.73
	PDW fL, median ± QD	16.10	0.15
PRP parameters	PLT 10^9^/L, median ± QD	343.00	65.00
	PCT ml/L, median ± QD	0.30	0.06
	MPV fL, median ± QD	8.60	0.40
	PDW fL, median ± QD	14.60	0.25

Legend: BMI, body mass index; MPV, mean platelet volume; PCT, plateletcrit; PDW, platelet distribution width; PLT, platelets; PRP, platelet-rich plasma; QD, quartile deviation; WB, whole blood.

**Table 2:** Frequency of genotypes and alleles of analyzed SNPs of the *VEGFA* gene.

SNP	Chromosome 6 Coordinate (GRCh37)	Genotypes	n (%)	Alleles	n (%)	*p* Value HWE Test
rs699947	43736389	AA	29 (21.97)	A	127 (48.11)	0.923
		AC	69 (52.27)	C	137 (51.89)	
		CC	34 (25.76)			
		AA + AC	98 (74.24)			
		AC + CC	103 (78.03)			
rs2010963	43738350	CC	10 (7.58)	C	76 (28.79)	0.978
		CG	56 (42.42)	G	188 (71.21)	
		GG	66 (50.00)			
		CC + CG	66 (50.00)			
		CG + GG	122 (92.42)			
rs1413711	43740678	CC	68 (51.51)	C	170 (64.39)	0.000
		CT	34 (25.76)	T	94 (35.61)	
		TT	30 (22.73)			
		CC + CT	102 (77.27)			
		CT + TT	64 (48.49)			
rs3024998	43745577	CC	65 (49.24)	C	187 (70.83)	0.916
		CT	57 (43.18)	T	77 (29.17)	
		TT	10 (7.58)			
		CC + CT	122 (92.42)			
		CT + TT	67 (50.76)			
rs3025021	43749163	CC	52 (39.39)	C	174 (65.91)	0.246
		CT	70 (53.03)	T	90 (34.09)	
		TT	10 (7.58)			
		CC + CT	122 (92.42)			
		CT + TT	80 (60.61)			

Legend: HWE, Hardy–Weinberg equilibrium; SNP, single nucleotide polymorphism.

**Table 3:** Frequency of haplotypes and diplotypes of the *VEGFA* gene in the study group.

SNP				Block Size (kb)	Frequency (%)
rs699947	rs2010963	rs1413711	rs3024998		
A	G	T	C	9	0.500
C	C	C	T		0.271
C	G	C	C		0.210
C	G	T	C		0.005
A	C	C	T		0.005
C	C	T	C		0.005
C	G	C	T		0.005
A	-	T	-	4	0.500
C	-	C	-		0.486
C	-	T	-		0.009
A	-	C	-		0.005
-	G	-	C	7	0.715
-	C	-	T		0.276
-	C	-	C		0.005
-	G	-	T		0.005

**Table 4:** Median (±QD) values of PROMs for genotypes of the *VEGFA* gene polymorphisms (additive model).

PROM	Week	Median ± QD in Respective Genotypes			*p* Value			
		rs2010963			Kruskal–Wallis	CC vs. CG	CC vs. GG	CG vs. GG
		CC	CG	GG				
QDASH	12	36.36 ± 18.18	30.68 ± 16.48	20.45 ± 20.45	0.029 *	1.000	1.000	0.024
	104	1.14 ± 25.00	22.73 ± 15.91	4.55 ± 12.50	0.017 *	0.477	1.000	0.018
ΔQDASH	12	15.91 ± 9.20	14.77 ± 15.34	27.27 ± 19.32	0.030 *	1.000	1.000	0.024
	104	27.27 ± 29.09	22.72 ± 16.92	40.90 ± 18.19	0.023 *	1.000	0.934	0.020
PRTEE	104	0.00 ± 21.75	14.75 ± 12.50	2.50 ± 8.25	0.022 *	0.510	1.000	0.025
			rs3024998		Kruskal–Wallis	CC vs. CT	CC vs. TT	CT vs. TT
		CC	CT	TT				
QDASH	12	22.73 ± 20.45	29.55 ± 17.05	36.36 ± 18.18	0.043 *	0.037	1.000	1.000
	104	4.55 ± 12.50	22.73 ± 19.32	1.14 ± 25.00	0.040 *	0.047	1.000	0.575
PRTEE	24	10.00 ± 17.25	20.00 ± 17.00	24.25 ± 20.50	0.048	0.043	1.000	1.000

Legend: QD, quartile deviation; PROM, patient-reported outcome measure; QDASH, quick version of disabilities of the arm, shoulder and hand score; PRTEE, patient-rated tennis elbow evaluation; *, differences remaining significant (*p* < 0.050) after removing diabetics from the analysis.

**Table 5:** Whole blood (WB) and platelet-rich plasma (PRP) parameter values with respect to the *VEGFA* gene polymorphisms variants (recessive/dominant model).

Rs Number	Parameter (Source)	Median	±QD	Median	±QD	*p*
rs2010963		GG		CC/CG		
	MPV fL (WB)	9.65	0.67	8.90	0.52	0.008
	EOS% (WB)	2.60	1.28	2.05	0.95	0.043
rs3024998		CC		CT/TT		
	PLT 10^9^/L (WB)	227.00	38.00	261.50	32.50	0.003
	MPV fL (WB)	9.70	0.65	8.90	0.50	0.003
	EOS% (WB)	2.60	1.35	1.90	0.90	0.024
	MPV fL (PRP)	8.60	0.50	8.40	0.50	0.027
	PDW fL (PRP)	14.60	0.20	14.50	0.20	0.030

Legend: EOS, eosinophils; MPV, mean platelet volume; PDW, platelet distribution width; PLT, platelets; PRP, platelet-rich plasma; QD, quartile deviation; WB, whole blood.

**Table 6:** Parameters differentiating genotypic variants of the rs2010963 and rs3024998 polymorphisms of the *VEGFA* gene in the recessive/dominant model.

**rs2010963**	**Parameter**	**GG**		**CC/CG**		***p***
		median	±QD	median	±QD	
	Alcohol units/week	1.00	4.00	0.00	2.00	0.023
		n	%	n	%	
	Cigarette smoking	16	24.24	6	9.09	0.036
	Diabetes mellitus	0	0.00	4	6.06	0.128
**rs3024998**	**Parameter**	**CC**		**CT/TT**		***p***
		median	±QD	median	±QD	
	Alcohol units/week	2.00	4.00	0.00	2.00	0.003
		n	%	n	%	
	Cigarette smoking	16	24.62	6	9.84	0.030
	Diabetes mellitus	0	0.00	4	5.97	0.136

Legend: QD, quartile deviation.

## Data Availability

Data are contained within the article and supplementary materials.

## Supplementary Materials

The supporting information can be downloaded at: https://www.mdpi.com/article/10.3390/ijms242417292/s1.

## References

[1] Millar N.L., Silbernagel K.G., Thorborg K., Kirwan P.D., Galatz L.M., Abrams G.D., Murrell G.A.C., McInnes I.B., Rodeo S.A. (2021). Tendinopathy. Nat. Rev. Dis. Prim..

[2] Keijsers R., de Vos R.J., Kuijer P.P.F., van den Bekerom M.P., van der Woude H.J., Eygendaal D. (2019). Tennis elbow. Shoulder Elb..

[3] Dean B.J.F., Gettings P., Dakin S.G., Carr A.J. (2016). Are inflammatory cells increased in painful human tendinopathy? A systematic review. Br. J. Sports Med..

[4] Melincovici C.S., Boşca A.B., Şuşman S., Mărginean M., Mihu C., Istrate M., Moldovan I.M., Roman A.L., Mihu C.M. (2018). Vascular endothelial growth factor (VEGF)—Key factor in normal and pathological angiogenesis. Rom. J. Morphol. Embryol..

[5] Kraus A., Sattler D., Wehland M., Luetzenberg R., Abuagela N., Infanger M. (2018). Vascular Endothelial Growth Factor Enhances Proliferation of Human Tenocytes and Promotes Tenogenic Gene Expression. Plast. Reconstr. Surg..

[6] Apte R.S., Chen D.S., Ferrara N. (2019). VEGF in Signaling and Disease: Beyond Discovery and Development. Cell.

[7] Matsumoto K., Ema M. (2014). Roles of VEGF-A signalling in development, regeneration, and tumours. J. Biochem..

[8] Vasta S., Di Martino A., Zampogna B., Torre G., Papalia R., Denaro V. (2016). Role of VEGF, Nitric Oxide, and Sympathetic Neurotransmitters in the Pathogenesis of Tendinopathy: A Review of the Current Evidences. Front. Aging Neurosci..

[9] Hu K., Olsen B.R. (2016). The roles of vascular endothelial growth factor in bone repair and regeneration. Bone.

[10] Liu X., Zhu B., Li Y., Liu X., Guo S., Wang C., Li S., Wang D. (2021). The Role of Vascular Endothelial Growth Factor in Tendon Healing. Front. Physiol..

[11] Riley G. (2008). Tendinopathy—From basic science to treatment. Nat. Clin. Pr. Rheumatol..

[12] Stevens A., Soden J., Brenchley P., Ralph S., Ray D.W. (2003). Haplotype analysis of the polymorphic human vascular endothelial growth factor gene promoter. Cancer Res..

[13] Shahbazi M., Fryer A.A., Pravica V., Brogan I.J., Ramsay H.M., Hutchinson I.V., Harden P.N. (2002). Vascular Endothelial Growth Factor Gene Polymorphisms Are Associated with Acute Renal Allograft Rejection. J. Am. Soc. Nephrol..

[14] Marsh S., Nakhoul F.M., Skorecki K., Rubin A., Miller B.P., Leibu R., Levy N.S., Levy A.P. (2000). Hypoxic induction of vascular endothelial growth factor is markedly decreased in diabetic individuals who do not develop retinopathy. Diabetes Care.

[15] Heist R.S., Zhai R., Liu G., Zhou W., Lin X., Su L., Asomaning K., Lynch T.J., Wain J.C., Christiani D.C. (2008). *VEGF* Polymorphisms and Survival in Early-Stage Non–Small-Cell Lung Cancer. J. Clin. Oncol..

[16] Masago K., Fujita S., Kim Y.H., Hatachi Y., Fukuhara A., Nagai H., Irisa K., Ichikawa M., Mio T., Mishima M. (2009). Effect of vascular endothelial growth factor polymorphisms on survival in advanced-stage non-small-cell lung cancer. Cancer Sci..

[17] Shadrina A.S., A Smetanina M., A Sokolova E., Shamovskaya D.V., Sevost‘ianova K.S., Shevela A.I., Soldatsky E.Y., Seliverstov E.I., Demekhova M.Y., A Shonov O. (2018). Allele rs2010963 C of the *VEGFA* gene is associated with the decreased risk of primary varicose veins in ethnic Russians. Phlebol. J. Venous Dis..

[18] Wang Y., Huang Q., Liu J., Wang Y., Zheng G., Lin L., Yu H., Tang W., Huang Z. (2017). Vascular endothelial growth factor A polymorphisms are associated with increased risk of coronary heart disease: A meta-analysis. Oncotarget.

[19] Moradzadegan A., Vaisi-Raygani A., Nikzamir A., Rahimi Z. (2015). Angiotensin converting enzyme insertion/deletion (I/D) (rs4646994) and Vegf polymorphism (+405G/C; rs2010963) in type II diabetic patients: Association with the risk of coronary artery disease. J. Renin-Angiotensin-Aldosterone Syst..

[20] Lulińska-Kuklik E., Leźnicka K., Humińska-Lisowska K., Moska W., Michałowska-Sawczyn M., Ossowski Z., Maculewicz E., Cięszczyk P., Kaczmarczyk M., Ratkowski W. (2019). The VEGFA gene and anterior cruciate ligament rupture risk in the Caucasian population. Biol. Sport.

[21] Rahim M., El Khoury L.Y., Raleigh S.M., Ribbans W.J., Posthumus M., Collins M., September A.V., Murthy K.R., Dammalli M., Pinto S.M. (2016). Human Genetic Variation, Sport and Exercise Medicine, and Achilles Tendinopathy: Role for Angiogenesis-Associated Genes. OMICS A J. Integr. Biol..

[22] Niemiec P., Szyluk K., Balcerzyk A., Kalita M., Jarosz A., Iwanicka J., Iwanicki T., Nowak T., Negru M., Francuz T. (2021). Why PRP works only on certain patients with tennis elbow? Is PDGFB gene a key for PRP therapy effectiveness? A prospective cohort study. BMC Musculoskelet. Disord..

[23] Szyluk K., Jarosz A., Balcerzyk-Matić A., Iwanicka J., Iwanicki T., Nowak T., Gierek M., Negru M., Kalita M., Górczyńska-Kosiorz S. (2022). Polymorphic Variants of the *PDGFRB* Gene Influence Efficacy of PRP Therapy in Treating Tennis Elbow: A Prospective Cohort Study. J. Clin. Med..

[24] Jarosz A., Szyluk K., Iwanicka J., Balcerzyk A., Nowak T., Iwanicki T., Negru M., Kalita M., Francuz T., Garczorz W. (2022). What Role Does *PDGFA* Gene Polymorphisms Play in Treating Tennis Elbow with PRP? A Prospective Cohort Study. J. Clin. Med..

[25] Chehadeh S.E., Sayed N.S., Abdelsamad H.S., Almahmeed W., Khandoker A.H., Jelinek H.F., Alsafar H.S. (2022). Genetic Variants and Their Associations to Type 2 Diabetes Mellitus Complications in the United Arab Emirates. Front. Endocrinol..

[26] Szwedowski D., Jaworski Ł., Szwedowska W., Pękala P., Gagat M. (2021). Neovascularization in Meniscus and Tendon Pathology as a Potential Mechanism in Regenerative Therapies: Special Reference to Platelet-Rich Plasma Treatment. Appl. Sci..

[27] Järvinen T.A. (2020). Neovascularisation in tendinopathy: From eradication to stabilisation?. Br. J. Sports Med..

[28] Chen C.-F., Liou S.-W., Wu H.-H., Lin C.-H., Huang L.-S., Woung L.-C., Tsai C.-Y. (2016). Regulatory SNPs Alter the Gene Expression of Diabetic Retinopathy Associated Secretary Factors. Int. J. Med. Sci..

[29] Di Stefano A.L., Labussiere M., Lombardi G., Eoli M., Bianchessi D., Pasqualetti F., Farina P., Cuzzubbo S., Gallego-Perez-Larraya J., Boisselier B. (2015). VEGFA SNP rs2010963 is associated with vascular toxicity in recurrent glioblastomas and longer response to bevacizumab. J. Neuro-Oncol..

[30] Ganapathy P., Sheshadri V.D.D., Sarkar R., Jones S., Gunasekaran K., Feyisa T.O., Umapathy D., Basha S. (2023). Vascular Endothelial Growth Factor Single Nucleotide Polymorphism +405 G/C (rs2010963) is associated with Levels, Infection Severity, and Amputation among South Indian Diabetic Foot Ulcer Patients. Evid.-Based Complement. Altern. Med..

[31] Watson C.J., Webb N.J., Bottomley M.J., Brenchley P.E. (2000). Identification of polymorphisms within the vascular endothelial growth factor (vegf) gene: Correlation with variation in vegf protein production. Cytokine.

[32] Sudhesan A., Rajappa M., Chandrashekar L., Ananthanarayanan P., Thappa D., Satheesh S., Chandrasekaran A. (2017). Vascular endothelial growth factor (VEGF) gene polymorphisms (rs699947, rs833061, and rs2010963) and psoriatic risk in South Indian Tamils. Hum. Immunol..

[33] Coskun S., Hirschi K.K. (2010). Establishment and regulation of the HSC niche: Roles of osteoblastic and vascular compartments. Birth Defects Res. Part C: Embryo Today Rev..

[34] Lombardi C., Berti A., Cottini M. (2022). The emerging roles of eosinophils: Implications for the targeted treatment of eosinophilic-associated inflammatory conditions. Curr. Res. Immunol..

[35] Gomułka K., Mędrala W. (2022). Serum Levels of Vascular Endothelial Growth Factor, Platelet Activating Factor and Eosinophil-Derived Neurotoxin in Chronic Spontaneous Urticaria—A Pilot Study in Adult Patients. Int. J. Mol. Sci..

[36] Lui P.P.Y. (2017). Tendinopathy in diabetes mellitus patients-Epidemiology, pathogenesis, and management. Scand. J. Med. Sci. Sports.

[37] Abate M., Schiavone C., Salini V., Andia I. (2013). Occurrence of tendon pathologies in metabolic disorders. Rheumatology.

[38] National Center for Biotechnology Information Database of Single Nucleotide Polymorphisms (dbSNP). https://www.ncbi.nlm.nih.gov/snp/.

[39] Machiela M.J., Chanock S.J. (2015). LDlink: A web-based application for exploring population-specific haplotype structure and linking correlated alleles of possible functional variants. Bioinformatics.

[40] Barrett J.C., Fry B., Maller J., Daly M.J. (2005). Haploview: Analysis and visualization of LD and haplotype maps. Bioinformatics.

[41] Gabriel S.B., Schaffner S.F., Nguyen H., Moore J.M., Roy J., Blumenstiel B., Higgins J., DeFelice M., Lochner A., Faggart M. (2002). The Structure of Haplotype Blocks in the Human Genome. Science.

[42] O’keeffe A.G., Ambler G., Barber J.A. (2017). Sample size calculations based on a difference in medians for positively skewed outcomes in health care studies. BMC Med. Res. Methodol..

