# Supplementary material for: Genetic Variability in VEGFA Gene Influences the Effectiveness of Tennis Elbow Therapy with PRP: A Two-Year Prospective Cohort Study
Source: Int J Mol Sci. 2023 Dec 9;24(24):17292. doi: 10.3390/ijms242417292 (PMC10743422; doi:10.3390/ijms242417292)
Supplement: Supplementary file 1 [file ijms-24-17292-s001.zip › ijms-2746118-supplementary.pdf]

Table S1. PROMs values in individuals with particular genotypes of the *VEGFA* gene polymorphisms in additive model.

PROM	week	Genotypes of rs699947						*P* value			
		AA		AC		CC					
		Median	±QD	Median	±QD	Median	±QD	Kruskal-Wallis test	AA vs AC	AA vs CC	AC vs CC
VAS	0	6.00	2.00	6.00	1.50	5.00	1.50	0.559			
	2	4.00	1.00	3.00	1.50	4.00	1.00	0.293			
	4	3.00	1.00	3.00	1.50	4.00	1.50	0.710			
	8	3.00	1.50	3.00	2.00	3.50	1.50	0.620			
	12	3.00	1.50	2.00	1.50	2.50	2.00	0.728			
	24	2.00	2.50	2.00	2.00	3.00	2.50	0.891			
	52	2.00	2.00	1.00	2.00	2.00	2.50	0.645			
	104	1.00	1.50	1.00	1.50	1.00	2.00	0.808			
ΔVAS (vs week 0)	2	1.00	1.50	2.00	1.50	1.00	1.50	0.595			
	4	3.00	1.25	2.00	1.75	1.50	2.00	0.095			
	8	3.00	2.00	2.00	2.00	2.00	2.00	0.249			
	12	3.00	1.75	2.00	2.00	2.50	2.50	0.334			
	24	3.00	1.50	2.00	2.00	2.50	2.50	0.513			
	52	4.00	2.50	4.00	2.00	2.00	2.50	0.686			
	104	4.50	2.25	4.00	2.00	4.00	2.50	0.908			
QDASH	0	56.81	12.37	50.00	15.91	52.27	12.50	0.336			
	2	36.36	14.77	39.77	16.48	39.77	11.36	0.612			
	4	36.36	11.36	31.82	16.48	38.64	12.50	0.293			
	8	31.82	19.32	29.55	17.05	40.91	18.18	0.109			
	12	27.27	21.59	25.00	14.77	34.09	15.91	0.316			
	24	14.77	22.16	23.86	20.45	34.09	23.86	0.307			
	52	22.73	21.59	13.64	19.89	31.82	26.14	0.272			
	104	6.82	21.59	11.36	13.64	20.91	28.41	0.633			
ΔQDASH (vs week 0)	2	6.81	13.07	5.69	13.64	6.81	16.57	0.828			
	4	19.31	12.50	11.36	15.91	11.36	15.91	0.646			
	8	19.32	21.02	15.91	18.19	11.36	14.41	0.680			
	12	19.32	22.09	18.18	18.18	18.18	10.23	0.661			
	24	20.45	18.18	21.59	20.46	17.04	20.46	0.521			
	52	18.17	18.19	30.68	19.31	15.91	21.59	0.588			
	104	32.95	18.75	29.55	22.73	28.41	21.14	0.832			
PRTEE	0	55.50	12.75	49.50	13.25	52.25	14.75	0.544			
	2	30.00	13.75	26.50	18.13	31.50	16.50	0.417			
	4	24.50	7.75	24.00	14.63	27.50	13.75	0.568			
	8	27.00	15.50	21.00	13.00	28.25	14.00	0.201			
	12	22.00	18.25	16.50	15.25	21.50	11.25	0.621			
	24	9.75	17.25	15.25	15.75	19.25	17.25	0.557			
	52	16.25	15.13	7.50	12.63	16.00	21.50	0.364			
	104	5.00	16.25	7.00	9.75	14.75	16.50	0.683			
ΔPRTEE (vs week 0)	2	17.50	10.75	16.00	11.88	12.50	13.75	0.619			
	4	29.00	13.88	21.25	11.13	19.75	14.75	0.332			
	8	31.25	16.63	28.00	15.25	17.75	15.50	0.203			
	12	32.75	20.63	29.50	17.00	25.25	12.00	0.565			

	24	32.50	17.50	28.50	19.50	26.50	15.50	0.295
	52	32.00	17.00	38.50	18.75	25.50	15.50	0.407
	104	37.25	18.38	38.50	16.25	37.75	18.50	0.707

**PROM**	**week**	**Genotypes of rs2010963**							***P* value**		
		**CC**		**CG**		**GG**					
		**Median**	**±QD**	**Median**	**±QD**	**Median**	**±QD**	**Kruskal-Wallis test**	**CC vs CG**	**CC vs GG**	**CG vs GG**
VAS	0	5.00	0.50	6.00	2.00	6.00	1.75	0.435			
	2	4.50	1.00	4.00	1.50	4.00	1.50	0.687			
	4	3.50	1.50	3.00	1.50	3.00	1.50	0.893			
	8	4.50	2.00	3.00	1.00	3.00	2.00	0.701			
	12	4.00	2.00	3.00	2.00	2.00	1.50	0.248			
	24	3.50	2.50	3.00	2.00	2.00	2.00	0.174			
	52	4.00	2.50	2.00	2.00	1.00	1.50	0.066			
	104	0.50	2.75	2.00	1.50	0.50	1.00	0.102			
ΔVAS (vs week 0)	2	0.00	1.50	1.00	2.00	1.00	1.50	0.165			
	4	0.50	1.50	2.00	2.00	2.75	1.50	0.424			
	8	0.50	1.50	2.00	2.50	3.00	1.50	0.304			
	12	2.50	1.50	2.00	2.50	3.00	1.50	0.216			
	24	1.50	2.00	2.00	2.00	3.00	1.50	0.092			
	52	1.00	2.00	2.00	3.50	4.00	2.00	0.030	0.577	0.064	0.208
	104	2.50	2.50	3.00	2.00	4.00	2.50	0.193			
QDASH	0	47.50	15.91	47.50	14.20	52.27	10.09	0.677			
	2	37.50	6.82	43.18	16.48	36.36	18.18	0.465			
	4	37.50	12.50	37.50	12.50	34.09	17.05	0.413			
	8	38.64	14.77	35.23	14.77	29.55	20.45	0.299			
	12	36.36	18.18	30.68	16.48	20.45	20.45	0.029	1.000	1.000	0.024
	24	39.77	26.14	31.82	15.91	15.91	22.73	0.084			
	52	39.77	26.14	20.45	21.59	13.64	22.73	0.432			
	104	1.14	25.00	22.73	15.91	4.55	12.50	0.017	0.477	1.000	0.018
ΔQDASH (vs week 0)	2	1.02	9.09	3.18	13.64	11.36	13.64	0.136			
	4	3.29	12.61	9.09	10.80	20.45	14.13	0.107			
	8	5.57	18.18	11.36	16.48	20.45	18.18	0.142			
	12	15.91	9.20	14.77	15.34	27.27	19.32	0.030	1.000	1.000	0.024
	24	12.50	16.02	13.64	20.45	28.36	18.75	0.041	1.000	0.460	0.053
	52	6.81	16.02	20.45	22.73	29.54	18.75	0.151			
	104	27.27	29.09	22.72	16.92	40.90	18.19	0.023	1.000	0.934	0.020
PRTEE	0	44.00	10.75	53.75	15.38	52.00	12.25	0.134			
	2	36.00	17.50	28.50	16.50	30.00	16.00	0.819			
	4	26.25	16.50	25.25	11.13	23.50	15.00	0.640			
	8	25.75	17.25	24.25	13.13	20.50	16.00	0.402			
	12	22.00	12.50	21.50	15.00	15.25	16.50	0.078			
	24	24.25	20.50	20.50	17.25	10.00	17.00	0.060			
	52	23.75	22.50	13.00	14.75	6.00	13.00	0.264			
	104	0.00	21.75	14.75	12.50	2.50	8.25	0.022	0.510	1.000	0.025
ΔPRTEE	2	10.25	10.75	14.50	13.88	17.50	10.38	0.117			
	4	5.75	12.50	20.50	13.13	26.45	12.88	0.150			

(vs week 0)	8	9.00	10.25	26.75	16.75	29.00	14.45	0.111			
	12	22.75	8.25	26.75	16.63	31.00	18.00	0.325			
	24	18.75	11.25	27.00	19.75	33.50	17.75	0.116			
	52	16.50	9.75	38.00	19.50	33.25	16.63	0.062			
	104	26.75	16.75	37.50	16.25	39.00	15.75	0.204			

**PROM**	**week**	**Genotypes of rs1413711**						***P* value**			
		**CC**		**CT**		**TT**					
		**Median**	**±QD**	**Median**	**±QD**	**Median**	**±QD**	**Kruskal-Wallis test**	**CC vs CT**	**CC vs TT**	**CT vs TT**
VAS	0	5.00	1.50	6.00	1.63	6.00	2.00	0.833			
	2	4.00	1.00	3.00	1.50	4.50	1.00	0.157			
	4	4.00	1.50	3.00	1.50	3.00	1.00	0.727			
	8	3.50	1.50	3.00	2.00	2.50	1.50	0.558			
	12	2.50	2.00	3.00	2.00	2.00	2.00	0.339			
	24	3.00	2.50	2.00	2.00	2.00	2.00	0.568			
	52	2.00	2.50	1.00	2.00	2.00	2.00	0.970			
	104	1.00	2.00	1.00	1.50	1.00	1.50	0.855			
ΔVAS	2	1.00	1.50	2.00	1.50	1.00	1.50	0.506			
(vs week 0)	4	1.50	2.00	2.00	1.50	3.00	1.50	0.290			
	8	2.00	2.00	2.50	2.00	3.00	2.00	0.358			
	12	2.50	2.50	2.00	2.00	3.00	1.50	0.330			
	24	2.50	2.50	2.00	2.00	3.00	1.50	0.469			
	52	2.00	2.50	4.00	2.00	4.00	2.25	0.659			
	104	4.00	2.50	4.00	2.00	5.00	2.00	0.975			
QDASH	0	52.27	12.50	52.27	16.48	52.27	11.37	0.469			
	2	39.77	11.36	38.64	15.91	44.32	15.91	0.335			
	4	38.64	12.50	29.55	15.91	36.36	9.09	0.207			
	8	40.91	18.18	29.55	17.61	29.55	19.32	0.121			
	12	34.09	15.91	27.27	17.61	21.59	20.45	0.179			
	24	34.09	23.86	25.00	20.45	13.64	20.45	0.194			
	52	31.82	26.14	13.64	21.59	20.45	18.18	0.349			
	104	20.91	28.41	14.77	14.78	6.82	21.59	0.606			
ΔQDASH	2	6.81	16.57	6.82	14.77	4.54	10.23	0.784			
(vs week 0)	4	11.36	15.91	11.36	15.91	17.27	11.36	0.979			
	8	11.36	14.41	15.91	18.75	18.18	18.18	0.700			
	12	18.18	10.23	18.18	18.75	25.00	20.45	0.448			
	24	17.04	20.46	20.45	19.32	23.86	16.54	0.344			
	52	15.91	21.59	27.27	19.32	21.59	16.48	0.631			
	104	28.41	21.14	29.54	22.73	34.09	18.18	0.788			
PRTEE	0	52.25	14.75	49.75	13.88	54.50	11.30	0.779			
	2	31.50	16.50	25.00	17.25	35.00	14.50	0.223			
	4	27.50	13.75	24.00	14.25	26.25	12.25	0.528			
	8	28.25	14.00	21.25	14.00	26.75	16.00	0.222			
	12	21.50	11.25	18.50	17.50	15.50	16.50	0.434			
	24	19.25	17.25	16.00	16.50	9.50	16.25	0.278			
	52	16.00	21.50	9.50	13.25	12.50	10.25	0.497			
	104	14.75	16.50	7.25	11.00	4.50	14.00	0.668			

ΔPRTEE (vs week 0)	2	12.50	13.75	17.50	13.00	17.00	8.75	0.693			
	4	19.75	14.75	21.50	12.25	27.00	10.50	0.527			
	8	17.75	15.50	26.75	15.50	31.00	15.00	0.205			
	12	25.25	12.00	28.00	17.13	36.50	15.75	0.234			
	24	26.50	15.50	28.50	19.75	34.75	14.50	0.132			
	52	25.50	15.50	36.00	19.00	32.75	17.13	0.332			
	104	37.75	18.50	38.00	16.75	39.00	16.75	0.599			

**PROM**	**week**	**Genotypes of rs3024998**						***P* value**			
		**CC**		**CT**		**TT**					
		**Median**	**±QD**	**Median**	**±QD**	**Median**	**±QD**	**Kruskal-Wallis test**	**CC vs CT**	**CC vs TT**	**CT vs TT**
VAS	0	6.00	1.75	6.00	2.00	5.00	0.50	0.422			
	2	4.00	1.50	4.00	1.50	4.50	1.00	0.611			
	4	3.00	1.50	3.00	1.50	3.50	1.50	0.952			
	8	3.00	2.00	3.00	1.00	4.50	2.00	0.727			
	12	2.00	1.50	3.00	2.00	4.00	2.00	0.266			
	24	1.50	2.00	2.50	2.00	3.50	2.50	0.138			
	52	1.00	1.50	2.00	2.00	4.00	2.50	0.074			
	104	1.00	1.50	2.00	1.50	0.50	2.75	0.253			
ΔVAS (vs week 0)	2	1.00	1.50	1.00	2.00	0.00	1.50	0.178			
	4	3.00	1.50	2.00	1.50	0.50	1.50	0.467			
	8	3.00	1.63	2.00	2.50	0.50	1.50	0.323			
	12	3.25	1.88	2.00	2.50	2.50	1.50	0.249			
	24	3.00	1.50	2.00	2.00	1.50	2.00	0.095			
	52	4.00	2.50	2.00	3.00	1.00	2.00	0.036	0.261	0.068	0.537
	104	4.25	2.50	3.00	2.00	2.50	2.50	0.243			
QDASH	0	52.27	10.09	47.72	13.64	47.50	15.91	0.751			
	2	36.36	17.78	43.18	15.91	37.50	6.82	0.849			
	4	36.36	17.61	34.09	11.36	37.50	12.50	0.669			
	8	29.55	20.45	34.09	15.91	38.64	14.77	0.403			
	12	22.73	20.45	29.55	17.05	36.36	18.18	0.043	0.037	1.000	1.000
	24	13.64	23.86	31.82	15.91	39.77	26.14	0.058			
	52	14.77	23.30	19.32	21.02	39.77	26.14	0.414			
	104	4.55	12.50	22.73	19.32	1.14	25.00	0.040	0.047	1.000	0.575
ΔQDASH (vs week 0)	2	11.36	12.50	4.54	12.59	1.02	9.09	0.442			
	4	18.18	14.64	9.09	13.63	3.29	12.61	0.292			
	8	19.32	18.75	11.36	18.18	5.57	18.18	0.233			
	12	26.13	20.45	15.90	14.77	15.91	9.20	0.059			
	24	27.27	19.31	14.77	20.43	12.50	16.02	0.043	0.056	0.457	1.000
	52	29.54	19.32	20.45	22.16	6.81	16.02	0.183			
	104	38.63	18.75	22.72	18.05	27.27	29.09	0.053			
PRTEE	0	53.00	12.25	53.50	15.50	44.00	10.75	0.160			
	2	33.25	16.38	28.00	15.75	36.00	17.50	0.964			
	4	24.50	17.13	24.50	11.00	26.25	16.50	0.868			
	8	21.00	16.00	22.00	13.25	25.75	17.25	0.537			
	12	15.50	16.50	21.50	14.75	22.00	12.50	0.106			
	24	10.00	17.25	20.00	17.00	24.25	20.50	0.048	0.043	1.000	1.000

PROM	week	Median	±QD	Median	±QD	Median	±QD	Kruskal-Wallis test	CC vs CT	CC vs TT	CT vs TT
	52	8.25	14.25	12.25	13.50	23.75	22.50	0.226			
	104	2.50	8.25	14.50	12.75	0.00	21.75	0.044	0.054	1.000	0.594
ΔPRTEE (vs week 0)	2	17.50	11.00	15.50	13.75	10.25	10.75	0.128			
	4	25.00	14.25	21.50	11.50	5.75	12.50	0.162			
	8	28.75	14.98	25.50	15.75	9.00	10.25	0.112			
	12	32.75	18.50	27.00	15.25	22.75	8.25	0.291			
	24	34.50	19.00	27.25	17.25	18.75	11.25	0.090			
	52	33.50	17.00	36.00	17.13	16.50	9.75	0.054			
	104	39.00	16.75	37.00	16.25	26.75	16.75	0.191			

**PROM**	**week**	**Genotypes of rs3025021**							***P* value**		
		**CC**		**CT**		**TT**					
		**Median**	**±QD**	**Median**	**±QD**	**Median**	**±QD**	**Kruskal-Wallis test**	**CC vs CT**	**CC vs TT**	**CT vs TT**
VAS	0	5.00	1.50	6.00	2.00	6.50	1.00	0.247			
	2	3.00	1.50	4.00	1.50	4.50	1.50	0.402			
	4	3.00	1.50	3.00	1.50	3.50	0.50	0.881			
	8	3.00	1.75	3.00	2.00	3.50	3.00	0.934			
	12	2.50	1.50	3.00	2.00	1.50	1.50	0.604			
	24	2.00	1.50	2.00	2.50	3.50	2.50	0.185			
	52	2.00	2.00	1.00	2.50	1.00	1.00	0.999			
	104	1.00	1.50	1.00	1.50	0.00	0.50	0.154			
ΔVAS (vs week 0)	2	1.00	1.50	1.00	2.00	1.50	2.50	0.946			
	4	2.25	1.50	2.00	2.00	3.00	2.50	0.686			
	8	3.00	2.00	2.00	2.50	4.00	3.00	0.698			
	12	3.00	1.50	2.00	2.00	4.50	3.50	0.714			
	24	3.00	1.50	2.00	2.00	2.50	3.00	0.839			
	52	4.00	2.00	3.00	2.50	5.00	2.50	0.790			
	104	4.00	1.50	4.00	2.50	6.00	1.00	0.157			
QDASH	0	47.72	12.51	52.27	12.50	52.27	15.91	0.586			
	2	34.09	14.77	43.18	17.05	47.73	17.05	0.402			
	4	34.09	14.77	37.50	14.77	32.95	11.36	0.899			
	8	29.55	18.75	34.09	17.05	29.55	19.32	0.694			
	12	26.14	16.48	30.68	20.45	25.00	14.77	0.512			
	24	22.73	21.59	31.82	21.59	25.00	15.91	0.724			
	52	20.45	21.59	20.45	25.00	11.36	9.09	0.813			
	104	15.91	17.05	13.64	22.73	5.68	6.82	0.546			
ΔQDASH (vs week 0)	2	5.68	12.50	6.81	15.91	10.22	15.91	0.949			
	4	11.36	11.36	13.63	17.05	6.82	20.32	0.858			
	8	13.63	15.92	15.91	19.32	15.90	29.41	0.884			
	12	20.45	15.91	17.07	15.91	23.86	29.55	0.959			
	24	18.18	17.04	20.45	22.73	18.17	6.82	0.905			
	52	28.36	17.05	20.45	26.14	21.59	18.18	0.871			
	104	29.54	10.23	29.55	22.73	42.04	18.18	0.731			
PRTEE	0	48.25	10.25	53.75	14.75	57.75	16.50	0.228			
	2	24.50	13.00	31.00	16.75	34.00	6.00	0.494			
	4	25.50	13.75	24.25	14.50	24.00	12.25	0.908			
	8	21.75	16.50	24.25	15.75	20.50	17.75	0.873			

	12	18.25	13.75	22.50	15.75	10.75	11.75	0.473
	24	13.50	16.75	15.00	18.50	14.25	15.75	0.789
	52	11.50	13.00	13.00	18.00	6.25	8.50	0.745
	104	7.50	11.75	9.00	14.00	1.50	3.50	0.559
ΔPRTEE (vs week 0)	2	17.00	9.75	13.75	14.50	17.50	16.50	0.597
	4	22.00	12.25	20.50	13.25	28.25	23.00	0.565
	8	24.50	15.00	28.00	14.75	44.00	34.50	0.398
	12	30.50	14.25	24.00	15.00	43.00	28.00	0.411
	24	30.75	14.13	28.00	21.25	34.75	13.75	0.703
	52	33.00	12.75	32.00	20.00	37.25	17.50	0.553
	104	37.25	13.75	38.00	18.75	50.50	16.25	0.199

Legend: *VEGFA*, vascular endothelial growth factor A; QD, Quartile Deviation; PROMs, patient-reported outcome measures; VAS, Visual Analog Scale; QDASH, quick version of Disabilities of the Arm, Shoulder and Hand score; PRTEE, Patient-Rated Tennis Elbow Evaluation.

Table S2. PROMs values in carriers of different genotypes of the rs699947 polymorphism of the *VEGFA* gene.

PROMs values in CC homozygotes and carriers of the A allele of the rs699947 *VEGFA* gene polymorphism.

**PROMs**		**CC rs699947**		**AC+AA rs699947**		***p***
	week	median	± QD	median	± QD	**Mann-Whitney U test**
VAS	0	5.00	1.50	6.00	1.75	0.631
	2	4.00	1.00	3.00	1.50	0.475
	4	4.00	1.50	3.00	1.50	0.436
	8	3.50	1.50	3.00	1.50	0.334
	12	2.50	2.00	2.50	1.50	0.703
	24	3.00	2.50	2.00	2.00	0.855
	52	2.00	2.50	1.00	2.00	0.807
	104	1.00	2.00	1.00	1.50	0.777
ΔVAS (vs week 0)	2	1.00	1.50	1.25	1.50	0.332
	4	1.50	2.00	2.00	1.50	0.287
	8	2.00	2.00	3.00	2.00	0.286
	12	2.50	2.50	3.00	2.00	0.574
	24	2.50	2.50	3.00	2.00	0.699
	52	2.00	2.50	4.00	2.00	0.387
	104	4.00	2.50	4.00	2.00	0.844
QDASH	0	52.27	12.50	52.27	14.77	0.233
	2	39.77	11.36	38.64	17.05	0.456
	4	38.64	12.50	34.09	15.91	0.143
	8	40.91	18.18	29.55	18.18	0.041
	12	34.09	15.91	27.27	17.05	0.153
	24	34.09	23.86	20.45	20.45	0.140
	52	31.82	26.14	14.77	21.02	0.148
	104	20.91	28.41	11.36	14.78	0.342
ΔQDASH (vs week 0)	2	6.81	16.57	6.81	13.63	0.628
	4	11.36	15.91	13.63	15.91	0.918
	8	11.36	14.41	15.91	18.19	0.411
	12	18.18	10.23	18.18	18.18	0.981
	24	17.04	20.46	20.45	19.32	0.544
	52	15.91	21.59	27.27	19.31	0.339
	104	28.41	21.14	31.81	22.73	0.570
PRTEE	0	52.25	14.75	52.00	13.25	0.934
	2	31.50	16.50	27.50	17.50	0.541
	4	27.50	13.75	24.50	13.75	0.371
	8	28.25	14.00	21.50	14.75	0.083
	12	21.50	11.25	17.25	16.50	0.331
	24	19.25	17.25	13.75	16.25	0.411
	52	16.00	21.50	10.00	13.13	0.239
	104	14.75	16.50	6.75	11.50	0.394
ΔPRTEE (vs week 0)	2	12.50	13.75	17.25	11.50	0.435
	4	19.75	14.75	22.00	11.75	0.391
	8	17.75	15.50	29.00	14.25	0.111
	12	25.25	12.00	29.50	18.50	0.659
	24	26.50	15.50	31.50	20.50	0.326
	52	25.50	15.50	33.50	18.75	0.181
	104	37.75	18.50	38.00	16.50	0.453

PROMs values in AA homozygotes and carriers of the C allele of the rs699947 *VEGFA* gene polymorphism.

**PROMs**		**AA rs699947**		**AC+CC rs699947**		***p***
	week	median	± QD	median	± QD	**Mann-Whitney U test**
VAS	0	6.00	2.00	6.00	1.50	0.287
	2	4.00	1.00	3.50	1.50	0.274
	4	3.00	1.00	3.00	1.50	0.615
	8	3.00	1.50	3.00	2.00	0.665
	12	3.00	1.50	2.00	2.00	0.435
	24	2.00	2.50	2.00	2.00	0.720
	52	2.00	2.00	1.00	2.00	0.436
	104	1.00	1.50	1.00	1.50	0.520
ΔVAS (vs week 0)	2	1.00	1.50	1.00	1.50	0.555
	4	3.00	1.25	2.00	2.00	0.033
	8	3.00	2.00	2.00	2.00	0.122
	12	3.00	1.75	2.00	2.50	0.141
	24	3.00	1.50	2.00	2.00	0.249
	52	4.00	2.50	3.25	2.00	0.809
	104	4.50	2.25	4.00	2.00	0.666
QDASH	0	56.81	12.37	52.27	13.64	0.654
	2	36.36	14.77	39.77	15.91	0.706
	4	36.36	11.36	36.36	15.91	0.951
	8	31.82	19.32	34.09	19.32	0.860
	12	27.27	21.59	29.55	18.18	0.355
	24	14.77	22.16	27.27	21.59	0.384
	52	22.73	21.59	15.91	23.86	0.822
	104	6.82	21.59	15.91	20.45	0.827
ΔQDASH (vs week 0)	2	6.81	13.07	6.81	14.77	0.841
	4	19.31	12.50	11.36	15.91	0.397
	8	19.32	21.02	13.63	17.16	0.588
	12	19.32	22.09	18.18	17.05	0.384
	24	20.45	18.18	20.45	19.89	0.269
	52	18.17	18.19	25.00	21.59	0.954
	104	32.95	18.75	29.54	23.73	0.713
PRTEE	0	55.50	12.75	50.50	13.25	0.285
	2	30.00	13.75	29.25	17.75	0.360
	4	24.50	7.75	24.75	14.75	0.799
	8	27.00	15.50	22.00	15.75	0.904
	12	22.00	18.25	20.00	14.50	0.713
	24	9.75	17.25	16.50	17.00	0.355
	52	16.25	15.13	11.25	15.50	0.701
	104	5.00	16.25	8.00	12.50	0.926
ΔPRTEE (vs week 0)	2	17.50	10.75	14.50	12.00	0.424
	4	29.00	13.88	20.50	13.00	0.157
	8	31.25	16.63	24.50	17.25	0.211
	12	32.75	20.63	27.00	15.25	0.290
	24	32.50	17.50	27.75	17.63	0.145
	52	32.00	17.00	33.75	18.00	0.724
	104	37.25	18.38	38.00	17.00	0.571

Legend: QD, Quartile Deviation; VAS, Visual Analog Scale; QDASH, quick version of Disabilities of the Arm, Shoulder and Hand score; PROM, Patient-Reported Outcome Measures; PRTEE, Patient-Rated Tennis Elbow Evaluation.

Table S3. PROMs values in carriers of different genotypes of the rs2010963 polymorphism of the *VEGFA* gene.

PROMs values in GG homozygotes and carriers of the C allele of the rs2010963 *VEGFA* gene polymorphism.

**PROMs**	week	**GG rs2010963** median	**GG rs2010963** ± QD	**CG+CC rs2010963** median	**CG+CC rs2010963** ± QD	***p*** **Mann-Whitney U test**
VAS	0	6.00	1.75	5.50	1.50	0.486
	2	4.00	1.50	4.00	1.50	0.961
	4	3.00	1.50	3.00	1.50	0.652
	8	3.00	2.00	3.00	1.50	0.559
	12	2.00	1.50	3.00	2.00	0.097
	24	2.00	2.00	3.00	2.00	0.071
	52	1.00	1.50	3.00	2.00	0.023 *
	104	0.50	1.00	2.00	1.50	0.039
ΔVAS (vs week 0)	2	1.00	1.50	1.00	1.50	0.364
	4	2.75	1.50	2.00	2.00	0.336
	8	3.00	1.50	2.00	2.00	0.341
	12	3.00	1.50	2.00	2.50	0.079
	24	3.00	1.50	2.00	2.50	0.039
	52	4.00	2.00	2.00	2.50	0.022 *
	104	4.00	2.50	3.00	2.50	0.102
QDASH	0	52.27	10.09	47.50	13.64	0.386
	2	36.36	18.18	43.18	15.91	0.249
	4	34.09	17.05	37.50	12.50	0.191
	8	29.55	20.45	36.36	14.77	0.124
	12	20.45	20.45	32.95	15.91	0.012 *
	24	15.91	22.73	31.82	21.59	0.030 *
	52	13.64	22.73	22.73	21.59	0.220
	104	4.55	12.50	22.73	21.59	0.013 *
ΔQDASH (vs week 0)	2	11.36	13.64	2.27	13.63	0.047
	4	20.45	14.13	7.95	10.23	0.035 *
	8	20.45	18.18	11.36	15.91	0.049
	12	27.27	19.32	14.77	14.77	0.010 *
	24	28.36	18.75	13.64	20.56	0.012 *
	52	29.54	18.75	15.91	21.59	0.087
	104	40.90	18.19	22.72	17.05	0.007 *
PRTEE	0	52.00	12.25	52.25	15.00	0.868
	2	30.00	16.00	29.25	16.50	0.531
	4	23.50	15.00	25.50	13.00	0.350
	8	20.50	16.00	24.25	13.75	0.188
	12	15.25	16.50	21.50	13.25	0.039 *
	24	10.00	17.00	20.50	17.75	0.029 *
	52	6.00	13.00	15.00	15.25	0.109
	104	2.50	8.25	14.25	13.50	0.017 *
ΔPRTEE (vs week 0)	2	17.50	10.38	14.00	13.50	0.320
	4	26.45	12.88	18.75	13.50	0.349
	8	29.00	14.45	24.75	17.25	0.400
	12	31.00	18.00	25.00	14.75	0.147
	24	33.50	17.75	25.00	17.25	0.082
	52	33.25	16.63	32.50	16.75	0.198
	104	39.00	15.75	36.25	16.00	0.201

***** **-** differences remaining significant ($p < 0.050$) after removing diabetics from the analysis.

PROMs values in CC homozygotes and carriers of the G allele of the rs2010963 *VEGFA* gene polymorphism.

**PROMs**	week	**CC rs2010963** median	**CC rs2010963** ± QD	**CG+GG rs2010963** median	**CG+GG rs2010963** ± QD	***p*** **Mann-Whitney U test**
VAS	0	5.00	0.50	6.00	2.00	0.217
	2	4.50	1.00	4.00	1.50	0.418
	4	3.50	1.50	3.00	1.50	0.790
	8	4.50	2.00	3.00	1.50	0.457
	12	4.00	2.00	2.00	1.50	0.738
	24	3.50	2.50	2.00	2.00	0.939
	52	4.00	2.50	1.00	2.00	0.251
	104	0.50	2.75	1.00	1.50	0.962
ΔVAS (vs week 0)	2	0.00	1.50	1.00	1.50	0.064
	4	0.50	1.50	2.00	1.50	0.263
	8	0.50	1.50	3.00	2.00	0.153
	12	2.50	1.50	3.00	2.00	0.527
	24	1.50	2.00	3.00	2.00	0.206
	52	1.00	2.00	4.00	2.50	0.056
	104	2.50	2.50	4.00	2.00	0.230
QDASH	0	47.50	15.91	52.27	13.64	0.695
	2	37.50	6.82	40.91	17.05	0.924
	4	37.50	12.50	36.36	14.77	0.561
	8	38.64	14.77	31.82	19.32	0.796
	12	36.36	18.18	27.27	17.05	0.921
	24	39.77	26.14	25.00	20.45	0.873
	52	39.77	26.14	15.91	22.73	0.455
	104	1.14	25.00	13.64	20.45	0.503
ΔQDASH (vs week 0)	2	1.02	9.09	6.81	13.64	0.717
	4	3.29	12.61	13.63	15.34	0.485
	8	5.57	18.18	15.91	18.18	0.469
	12	15.91	9.20	20.45	17.05	0.914
	24	12.50	16.02	20.45	18.19	0.388
	52	6.81	16.02	22.73	19.32	0.169
	104	27.27	29.09	31.81	22.73	0.670
PRTEE	0	44.00	10.75	53.25	14.25	0.063
	2	36.00	17.50	29.00	16.00	0.805
	4	26.25	16.50	24.50	13.00	0.700
	8	25.75	17.25	22.00	15.00	0.511
	12	22.00	12.50	19.75	15.50	0.780
	24	24.25	20.50	14.75	16.25	0.820
	52	23.75	22.50	11.50	14.13	0.455
	104	0.00	21.75	7.50	12.63	0.505
ΔPRTEE (vs week 0)	2	10.25	10.75	16.75	12.88	0.044
	4	5.75	12.50	21.75	13.13	0.057
	8	9.00	10.25	28.00	15.45	0.038
	12	22.75	8.25	29.00	17.50	0.446
	24	18.75	11.25	31.50	19.25	0.113
	52	16.50	9.75	34.00	18.75	0.024
	104	26.75	16.75	38.50	16.50	0.123

Legend: QD, Quartile Deviation; VAS, Visual Analog Scale; QDASH, quick version of Disabilities of the Arm, Shoulder and Hand score; PROM, Patient-Reported Outcome Measures; PRTEE, Patient-Rated Tennis Elbow Evaluation.

Table S4. PROMs values in carriers of different genotypes of the rs1413711 polymorphism of the *VEGFA* gene.

PROMs values in TT homozygotes and carriers of the C allele of the rs1413711 *VEGFA* gene polymorphism.

**PROMs**		**TT rs1413711**		**CT+CC rs1413711**		***p***
	week	median	± QD	median	± QD	**Mann-Whitney U test**
VAS	0	6.00	2.00	6.00	1.50	0.618
	2	4.50	1.00	3.00	1.50	0.145
	4	3.00	1.00	3.00	1.50	0.687
	8	2.50	1.50	3.00	2.00	0.447
	12	2.00	2.00	3.00	2.00	0.143
	24	2.00	2.00	2.00	2.00	0.354
	52	2.00	2.00	2.00	2.00	0.936
	104	1.00	1.50	1.00	1.50	0.586
ΔVAS (vs week 0)	2	1.00	1.50	1.00	1.50	0.766
	4	3.00	1.50	2.00	2.00	0.152
	8	3.00	2.00	2.00	2.00	0.215
	12	3.00	1.50	2.00	2.00	0.138
	24	3.00	1.50	2.00	2.00	0.219
	52	4.00	2.25	3.00	2.00	0.587
	104	5.00	2.00	4.00	2.00	0.878
QDASH	0	52.27	11.37	52.27	13.64	0.918
	2	44.32	15.91	38.64	14.77	0.336
	4	36.36	9.09	34.09	14.77	0.637
	8	29.55	19.32	34.09	19.32	0.619
	12	21.59	20.45	29.55	19.32	0.116
	24	13.64	20.45	27.27	21.59	0.142
	52	20.45	18.18	18.18	22.73	0.589
	104	6.82	21.59	17.04	20.45	0.552
ΔQDASH (vs week 0)	2	4.54	10.23	6.81	14.77	0.531
	4	17.27	11.36	11.36	15.91	0.898
	8	18.18	18.18	14.77	17.16	0.663
	12	25.00	20.45	18.18	17.05	0.227
	24	23.86	16.54	20.45	20.45	0.147
	52	21.59	16.48	22.72	21.59	0.740
	104	34.09	18.18	29.54	23.73	0.586
PRTEE	0	54.50	11.30	50.75	14.50	0.490
	2	35.00	14.50	28.00	16.25	0.183
	4	26.25	12.25	24.50	14.25	0.722
	8	26.75	16.00	22.00	15.75	0.589
	12	15.50	16.50	20.50	14.25	0.265
	24	9.50	16.25	18.00	17.50	0.118
	52	12.50	10.25	11.50	15.75	0.651
	104	4.50	14.00	8.25	12.75	0.595
ΔPRTEE (vs week 0)	2	17.00	8.75	14.50	13.00	0.942
	4	27.00	10.50	20.50	13.25	0.333
	8	31.00	15.00	24.50	17.25	0.211
	12	36.50	15.75	26.75	15.50	0.089
	24	34.75	14.50	27.50	17.50	0.048
	52	32.75	17.13	33.00	18.00	0.304
	104	39.00	16.75	38.00	17.00	0.381

PROMs values in CC homozygotes and carriers of the T allele of thers1413711 *VEGFA* gene polymorphism.

**PROMs**		**CC rs1413711**		**CT+TT rs1413711**		***p***
	week	median	± QD	median	± QD	**Mann-Whitney U test**
VAS	0	5.00	1.50	6.00	1.75	0.631
	2	4.00	1.00	3.00	1.50	0.475
	4	4.00	1.50	3.00	1.50	0.436
	8	3.50	1.50	3.00	1.50	0.334
	12	2.50	2.00	2.50	1.50	0.703
	24	3.00	2.50	2.00	2.00	0.855
	52	2.00	2.50	1.00	2.00	0.807
	104	1.00	2.00	1.00	1.50	0.777
ΔVAS (vs week 0)	2	1.00	1.50	1.25	1.50	0.332
	4	1.50	2.00	2.00	1.50	0.287
	8	2.00	2.00	3.00	2.00	0.286
	12	2.50	2.50	3.00	2.00	0.574
	24	2.50	2.50	3.00	2.00	0.699
	52	2.00	2.50	4.00	2.00	0.387
	104	4.00	2.50	4.00	2.00	0.844
QDASH	0	52.27	12.50	52.27	14.77	0.233
	2	39.77	11.36	38.64	17.05	0.456
	4	38.64	12.50	34.09	15.91	0.143
	8	40.91	18.18	29.55	18.18	0.041
	12	34.09	15.91	27.27	17.05	0.153
	24	34.09	23.86	20.45	20.45	0.140
	52	31.82	26.14	14.77	21.02	0.148
	104	20.91	28.41	11.36	14.78	0.342
ΔQDASH (vs week 0)	2	6.81	16.57	6.81	13.63	0.628
	4	11.36	15.91	13.63	15.91	0.918
	8	11.36	14.41	15.91	18.19	0.411
	12	18.18	10.23	18.18	18.18	0.981
	24	17.04	20.46	20.45	19.32	0.544
	52	15.91	21.59	27.27	19.31	0.339
	104	28.41	21.14	31.81	22.73	0.570
PRTEE	0	52.25	14.75	52.00	13.25	0.934
	2	31.50	16.50	27.50	17.50	0.541
	4	27.50	13.75	24.50	13.75	0.371
	8	28.25	14.00	21.50	14.75	0.083
	12	21.50	11.25	17.25	16.50	0.331
	24	19.25	17.25	13.75	16.25	0.411
	52	16.00	21.50	10.00	13.13	0.239
	104	14.75	16.50	6.75	11.50	0.394
ΔPRTEE (vs week 0)	2	12.50	13.75	17.25	11.50	0.435
	4	19.75	14.75	22.00	11.75	0.391
	8	17.75	15.50	29.00	14.25	0.111
	12	25.25	12.00	29.50	18.50	0.659
	24	26.50	15.50	31.50	20.50	0.326
	52	25.50	15.50	33.50	18.75	0.181
	104	37.75	18.50	38.00	16.50	0.453

Legend: QD, Quartile Deviation; VAS, Visual Analog Scale; QDASH, quick version of Disabilities of the Arm, Shoulder and Hand score; PROM, Patient-Reported Outcome Measures; PRTEE, Patient-Rated Tennis Elbow Evaluation.

Table S5. PROMs values in carriers of different genotypes of the rs3024998 polymorphism of the *VEGFA* gene.

PROMs values in TT homozygotes and carriers of the C allele of the rs3024998 *VEGFA* gene polymorphism.

**PROMs**		**TT rs3024998**		**CT+CC rs3024998**		***p***
	week	median	± QD	median	± QD	**Mann-Whitney U test**
VAS	0	5.00	0.50	6.00	2.00	0.217
	2	4.50	1.00	4.00	1.50	0.418
	4	3.50	1.50	3.00	1.50	0.790
	8	4.50	2.00	3.00	1.50	0.457
	12	4.00	2.00	2.00	1.50	0.738
	24	3.50	2.50	2.00	2.00	0.939
	52	4.00	2.50	1.00	2.00	0.251
	104	0.50	2.75	1.00	1.50	0.962
ΔVAS (vs week 0)	2	0.00	1.50	1.00	1.50	0.064
	4	0.50	1.50	2.00	1.50	0.263
	8	0.50	1.50	3.00	2.00	0.153
	12	2.50	1.50	3.00	2.00	0.527
	24	1.50	2.00	3.00	2.00	0.206
	52	1.00	2.00	4.00	2.50	0.056
	104	2.50	2.50	4.00	2.00	0.230
QDASH	0	47.50	15.91	52.27	13.64	0.695
	2	37.50	6.82	40.91	17.05	0.924
	4	37.50	12.50	36.36	14.77	0.561
	8	38.64	14.77	31.82	19.32	0.796
	12	36.36	18.18	27.27	17.05	0.921
	24	39.77	26.14	25.00	20.45	0.873
	52	39.77	26.14	15.91	22.73	0.455
	104	1.14	25.00	13.64	20.45	0.503
ΔQDASH (vs week 0)	2	1.02	9.09	6.81	13.64	0.717
	4	3.29	12.61	13.63	15.34	0.485
	8	5.57	18.18	15.91	18.18	0.469
	12	15.91	9.20	20.45	17.05	0.914
	24	12.50	16.02	20.45	18.19	0.388
	52	6.81	16.02	22.73	19.32	0.169
	104	27.27	29.09	31.81	22.73	0.670
PRTEE	0	44.00	10.75	53.25	14.25	0.063
	2	36.00	17.50	29.00	16.00	0.805
	4	26.25	16.50	24.50	13.00	0.700
	8	25.75	17.25	22.00	15.00	0.511
	12	22.00	12.50	19.75	15.50	0.780
	24	24.25	20.50	14.75	16.25	0.820
	52	23.75	22.50	11.50	14.13	0.455
	104	0.00	21.75	7.50	12.63	0.505
ΔPRTEE (vs week 0)	2	10.25	10.75	16.75	12.88	0.044
	4	5.75	12.50	21.75	13.13	0.057
	8	9.00	10.25	28.00	15.45	0.038
	12	22.75	8.25	29.00	17.50	0.446
	24	18.75	11.25	31.50	19.25	0.113
	52	16.50	9.75	34.00	18.75	0.024
	104	26.75	16.75	38.50	16.50	0.123

PROMs values in CC homozygotes and carriers of the T allele of the rs3024998 *VEGFA* gene polymorphism.

**PROMs**	week	**CC rs3024998** median	**CC rs3024998** ± QD	**CT+TTrs3024998** median	**CT+TTrs3024998** ± QD	***p*** **Mann-Whitney U test**
VAS	0	6.00	1.75	5.00	1.50	0.444
	2	4.00	1.50	4.00	1.50	0.755
	4	3.00	1.50	3.00	1.50	0.824
	8	3.00	2.00	3.00	1.50	0.634
	12	2.00	1.50	3.00	2.00	0.105
	24	1.50	2.00	3.00	2.00	0.054
	52	1.00	1.50	2.50	2.00	0.027 *
	104	1.00	1.50	1.00	1.50	0.109
ΔVAS (vs week 0)	2	1.00	1.50	1.00	1.50	0.553
	4	3.00	1.50	2.00	2.00	0.422
	8	3.00	1.63	2.00	2.00	0.404
	12	3.25	1.88	2.00	2.50	0.098
	24	3.00	1.50	2.00	2.25	0.040
	52	4.00	2.50	2.00	2.50	0.028 *
	104	4.25	2.50	3.00	2.50	0.145
QDASH	0	52.27	10.09	47.72	13.64	0.466
	2	36.36	17.78	43.18	14.77	0.609
	4	36.36	17.61	36.36	12.50	0.416
	8	29.55	20.45	36.36	15.91	0.180
	12	22.73	20.45	31.82	15.91	0.018 *
	24	13.64	23.86	31.82	18.75	0.020 *
	52	14.77	23.30	21.59	21.59	0.207
	104	4.55	12.50	22.73	21.59	0.031 *
ΔQDASH (vs week 0)	2	11.36	12.50	2.27	12.59	0.202
	4	18.18	14.64	9.09	13.64	0.123
	8	19.32	18.75	11.36	18.18	0.092
	12	26.13	20.45	15.90	14.77	0.021 *
	24	27.27	19.31	14.77	19.93	0.012 *
	52	29.54	19.32	17.04	22.73	0.119
	104	38.63	18.75	22.72	18.18	0.016 *
PRTEE	0	53.00	12.25	51.50	15.50	0.908
	2	33.25	16.38	28.00	17.00	0.978
	4	24.50	17.13	25.00	11.75	0.648
	8	21.00	16.00	24.00	14.50	0.296
	12	15.50	16.50	21.50	13.50	0.053 (0.047)*
	24	10.00	17.25	20.00	17.13	0.023 *
	52	8.25	14.25	14.50	15.25	0.089
	104	2.50	8.25	14.00	14.00	0.034 *
ΔPRTEE (vs week 0)	2	17.50	11.00	14.50	13.75	0.678
	4	25.00	14.25	20.50	13.50	0.513
	8	28.75	14.98	24.50	17.25	0.435
	12	32.75	18.50	26.50	14.25	0.126
	24	34.50	19.00	25.50	16.25	0.056
	52	33.50	17.00	32.25	16.50	0.150
	104	39.00	16.75	36.50	15.50	0.180

***** **-** differences significant ($p < 0.050$) after removing diabetics from the analysis.

Legend: QD, Quartile Deviation; VAS, Visual Analog Scale; QDASH, quick version of Disabilities of the Arm, Shoulder and Hand score; PROM, Patient-Reported Outcome Measures; PRTEE, Patient-Rated Tennis Elbow Evaluation.

Table S6. PROMs values in carriers of different genotypes of the rs3025021 polymorphism of the *VEGFA* gene.

PROMs values in TT homozygotes and carriers of the C allele of the rs3025021 *VEGFA* gene polymorphism.

**PROMs**	week	**TT rs3025021** median	**TT rs3025021** ± QD	**CT+CC rs3025021** median	**CT+CC rs3025021** ± QD	***p*** **Mann-Whitney U test**
VAS	0	6.50	1.00	6.00	1.50	0.319
	2	4.50	1.50	4.00	1.50	0.255
	4	3.50	0.50	3.00	1.50	0.934
	8	3.50	3.00	3.00	2.00	0.718
	12	1.50	1.50	3.00	1.50	0.602
	24	3.50	2.50	2.00	2.00	0.070
	52	1.00	1.00	2.00	2.00	0.986
	104	0.00	0.50	1.00	1.50	0.081
ΔVAS (vs week 0)	2	1.50	2.50	1.00	1.50	0.747
	4	3.00	2.50	2.00	2.00	0.390
	8	4.00	3.00	2.00	2.00	0.663
	12	4.50	3.50	3.00	2.00	0.475
	24	2.50	3.00	3.00	2.00	0.558
	52	5.00	2.50	3.00	2.00	0.507
	104	6.00	1.00	4.00	2.00	0.061
QDASH	0	52.27	15.91	52.27	12.50	0.870
	2	47.73	17.05	38.64	15.91	0.811
	4	32.95	11.36	36.36	14.77	0.965
	8	29.55	19.32	34.09	19.32	0.826
	12	25.00	14.77	29.55	17.05	0.757
	24	25.00	15.91	25.00	22.73	0.566
	52	11.36	9.09	20.45	23.86	0.556
	104	5.68	6.82	15.91	21.59	0.281
ΔQDASH (vs week 0)	2	10.22	15.91	6.81	13.64	0.889
	4	6.82	20.32	12.50	15.34	0.830
	8	15.90	29.41	15.90	17.05	0.941
	12	23.86	29.55	18.18	15.91	0.808
	24	18.17	6.82	20.45	19.32	0.710
	52	21.59	18.18	22.72	21.59	0.603
	104	42.04	18.18	29.55	22.72	0.445
PRTEE	0	57.75	16.50	51.50	13.25	0.280
	2	34.00	6.00	29.00	16.00	0.842
	4	24.00	12.25	25.00	14.00	0.742
	8	20.50	17.75	22.00	15.75	0.737
	12	10.75	11.75	20.50	15.00	0.579
	24	14.25	15.75	14.75	17.25	0.575
	52	6.25	8.50	12.25	15.63	0.979
	104	1.50	3.50	8.00	14.00	0.283
ΔPRTEE (vs week 0)	2	17.50	16.50	14.50	11.50	0.385
	4	28.25	23.00	21.00	13.00	0.345
	8	44.00	34.50	25.50	14.50	0.372
	12	43.00	28.00	27.00	15.25	0.189
	24	34.75	13.75	30.50	18.00	0.420
	52	37.25	17.50	32.50	18.00	0.287
	104	50.50	16.25	37.50	16.50	0.079

PROMs values in CC homozygotes and carriers of the T allele of the rs3025021 *VEGFA* gene polymorphism.

**PROMs**		**CC rs3025021**		**CT+TT rs3025021**		***p***
	week	median	± QD	median	± QD	**Mann-Whitney U test**
VAS	0	5.00	1.50	6.00	2.00	0.125
	2	3.00	1.50	4.00	1.50	0.338
	4	3.00	1.50	3.00	1.50	0.617
	8	3.00	1.75	3.00	2.00	0.976
	12	2.50	1.50	2.50	1.75	0.479
	24	2.00	1.50	2.00	2.00	0.494
	52	2.00	2.00	1.00	2.50	0.977
	104	1.00	1.50	1.00	1.50	0.230
ΔVAS (vs week 0)	2	1.00	1.50	1.00	2.00	0.994
	4	2.25	1.50	2.00	2.00	0.770
	8	3.00	2.00	2.00	2.50	0.421
	12	3.00	1.50	2.00	2.00	0.829
	24	3.00	1.50	2.00	2.00	0.847
	52	4.00	2.00	3.00	2.50	0.758
	104	4.00	1.50	4.00	2.50	0.410
QDASH	0	47.72	12.51	52.27	13.07	0.342
	2	34.09	14.77	43.18	17.05	0.178
	4	34.09	14.77	36.36	14.77	0.664
	8	29.55	18.75	34.09	17.05	0.454
	12	26.14	16.48	29.55	19.32	0.313
	24	22.73	21.59	27.27	20.46	0.500
	52	20.45	21.59	18.18	25.00	0.920
	104	15.91	17.05	11.36	21.59	0.663
ΔQDASH (vs week 0)	2	5.68	12.50	6.81	15.34	0.756
	4	11.36	11.36	13.63	17.05	0.658
	8	13.63	15.92	15.91	19.94	0.622
	12	20.45	15.91	17.07	19.72	0.929
	24	18.18	17.04	20.45	21.59	0.881
	52	28.36	17.05	20.45	25.00	0.868
	104	29.54	10.23	31.82	22.73	0.718
PRTEE	0	48.25	10.25	54.00	15.13	0.122
	2	24.50	13.00	32.00	16.50	0.237
	4	25.50	13.75	24.25	14.50	0.841
	8	21.75	16.50	24.25	15.88	0.762
	12	18.25	13.75	21.50	15.38	0.354
	24	13.50	16.75	15.00	18.50	0.609
	52	11.50	13.00	13.00	17.50	0.453
	104	7.50	11.75	7.00	12.75	0.779
ΔPRTEE (vs week 0)	2	17.00	9.75	14.25	14.50	0.761
	4	22.00	12.25	21.00	15.00	0.488
	8	24.50	15.00	28.00	16.63	0.232
	12	30.50	14.25	26.75	17.38	0.917
	24	30.75	14.13	30.50	21.50	0.691
	52	33.00	12.75	33.00	19.50	0.961
	104	37.25	13.75	38.00	18.00	0.459

Legend: QD, Quartile Deviation; VAS, Visual Analog Scale; QDASH, quick version of Disabilities of the Arm, Shoulder and Hand score; PROM, Patient-Reported Outcome Measures; PRTEE, Patient-Rated Tennis Elbow Evaluation.

Table S7. Whole blood (WB) and platelet-rich plasma (PRP) parameters values in individuals with particular genotypes of the *VEGFA* gene polymorphisms (additive model).

Parameter	Source	Genotypes of rs699947						*P* value			
		AA		AC		CC					
		Median	±QD	Median	±QD	Median	±QD	Kruskal-Wallis test	AA vs AC	AA vs CC	AC vs CC
PLT 10^9/l	WB	221.50	38.00	238.00	39.00	264.00	28.00	0.019	0.069	0.022	1.000
PCT ml/l	WB	2.04	0.31	2.33	0.39	2.35	0.28	0.107			
MPV fl	WB	9.70	0.70	9	0.70	8.9	0.55	0.041			
PDW fl	WB	16.15	0.25	16.1	0.15	16	0.15	0.641			
WBC 10^9/l	WB	5.38	1.01	6.28	1.05	6.8	1.15	0.044	0.181	0.043	1.000
RBC 10^{12}/l	WB	4.73	0.41	4.68	0.27	4.54	0.27	0.281			
EOS%	WB	2.60	1.00	2.3	1.05	2.1	0.70	0.266			
EOS# 10*9/L	WB	0.14	0.04	0.14	0.08	0.14	0.06	0.942			
PLT 10^9/l	PRP	349.00	84.00	335	50.50	351.5	54.50	0.479			
PCT ml/l	PRP	0.31	0.07	0.285	0.07	0.31	0.05	0.287			
MPV fl	PRP	8.80	0.55	8.5	0.40	8.7	0.40	0.028	0.023	0.281	1.000
PDW fl	PRP	14.70	0.25	14.5	0.20	14.6	0.25	0.089			

Parameter	Source	Genotypes of rs2010963						*P* value			
		CC		CG		GG					
		Median	±QD	Median	±QD	Median	±QD	Kruskal-Wallis test	CC vs CG	CC vs GG	CG vs GG
PLT 10^9/l	WB	266.00	22.75	251.50	32.00	227.00	38.25	0.197			
PCT ml/l	WB	2.39	0.34	2.32	0.37	2.15	0.37	0.620			
MPV fl	WB	8.75	0.42	8.90	0.70	9.65	0.67	0.018	0.942	0.079	0.075
PDW fl	WB	16.00	0.07	16.10	0.20	16.10	0.15	0.375			
WBC 10^9/l	WB	8.03	2.71	6.30	0.97	6.07	1.17	0.028	0.035	0.025	1.000
RBC 10^{12}/l	WB	4.72	0.13	4.56	0.29	4.68	0.29	0.680			
EOS%	WB	2.10	0.58	1.90	1.08	2.60	1.28	0.121			
EOS# 10*9/L	WB	0.17	0.04	0.12	0.06	0.16	0.07	0.180			
PLT 10^9/l	PRP	375.00	51.00	339.50	54.50	345.00	77.50	0.940			
PCT ml/l	PRP	0.31	0.05	0.30	0.05	0.30	0.06	0.995			
MPV fl	PRP	8.30	0.40	8.50	0.45	8.60	0.50	0.111			
PDW fl	PRP	14.45	0.25	14.50	0.20	14.60	0.20	0.384			

Parameter	Source	Genotypes of rs1413711						P value			
		CC		CT		TT					
		**Median**	**±QD**	**Median**	**±QD**	**Median**	**±QD**	**Kruskal-Wallis test**	**CC vs CT**	**CC vs TT**	**CT vs TT**
PLT 10^9/l	WB	264.00	28.00	242.00	37.75	220.00	37.00	0.009	1.000	0.012	0.030
PCT ml/l	WB	2.35	0.28	2.33	0.38	2.03	0.31	0.090			
MPV fl	WB	8.90	0.55	9.00	0.68	9.70	0.65	0.018	1.000	0.019	0.079
PDW fl	WB	16.00	0.15	16.05	0.15	16.20	0.25	0.444			
WBC 10^9/l	WB	6.80	1.15	6.32	1.07	5.29	0.79	0.023	1.000	0.025	0.086
RBC 10^{12}/l	WB	4.54	0.27	4.68	0.26	4.75	0.41	0.262			
EOS%	WB	2.10	0.70	2.30	1.03	2.60	1.00	0.306			
EOS# 10*9/L	WB	0.14	0.06	0.14	0.08	0.13	0.04	0.990			
PLT 10^9/l	PRP	351.50	54.50	337.00	60.00	343.50	83.50	0.571			
PCT ml/l	PRP	0.31	0.05	0.29	0.07	0.30	0.06	0.436			
MPV fl	PRP	8.70	0.40	8.50	0.35	8.80	0.60	0.054			
PDW fl	PRP	14.60	0.25	14.50	0.20	14.75	0.30	0.033	0.691	0.639	0.031

Parameter	Source	Genotypes of rs3024998						P value			
		CC		CT		TT					
		**Median**	**±QD**	**Median**	**±QD**	**Median**	**±QD**	**Kruskal-Wallis test**	**CC vs CT**	**CC vs TT**	**CT vs TT**
PLT 10^9/l	WB	227.00	38.00	253.00	34.50	266.00	22.75	0.053			
PCT ml/l	WB	2.10	0.32	2.34	0.38	2.39	0.34	0.255			
MPV fl	WB	9.70	0.65	8.90	0.55	8.75	0.42	0.008	0.030	0.061	1.000
PDW fl	WB	16.10	0.15	16.10	0.25	16.00	0.07	0.341			
WBC 10^9/l	WB	5.98	1.25	6.28	0.97	8.03	2.71	0.027	1.000	0.025	0.035
RBC 10^{12}/l	WB	4.69	0.30	4.54	0.28	4.72	0.13	0.358			
EOS%	WB	2.60	1.35	1.80	1.00	2.10	0.58	0.075			
EOS# 10*9/L	WB	0.17	0.07	0.12	0.05	0.17	0.04	0.106			
PLT 10^9/l	PRP	349.00	72.50	339.00	66.00	375.00	51.00	0.806			
PCT ml/l	PRP	0.30	0.06	0.29	0.06	0.31	0.05	0.926			
MPV fl	PRP	8.60	0.50	8.50	0.50	8.30	0.40	0.055			
PDW fl	PRP	14.60	0.20	14.50	0.25	14.45	0.25	0.088			

Parameter	Source	Genotypes of rs3025021						*P* value			
		CC		CT		TT					
		Median	±QD	Median	±QD	Median	±QD	Kruskal-Wallis test	CC vs CT	CC vs TT	CT vs TT
PLT 10^9/l	WB	236.50	41.50	246.00	41.00	227.00	9.50	0.756			
PCT ml/l	WB	2.21	0.39	2.32	0.37	2.31	0.16	0.918			
MPV fl	WB	9.05	0.75	9.40	0.70	9.10	0.75	0.933			
PDW fl	WB	16.05	0.20	16.10	0.20	16.00	0.10	0.622			
WBC 10^9/l	WB	6.25	1.57	6.36	1.04	5.13	0.59	0.214			
RBC 10^{12}/l	WB	4.66	0.31	4.67	0.35	4.75	0.21	0.795			
EOS%	WB	1.90	0.65	2.50	1.05	2.00	1.40	0.258			
EOS# 10*9/L	WB	0.12	0.05	0.17	0.08	0.16	0.05	0.270			
PLT 10^9/l	PRP	356.50	74.00	334.00	59.25	344.00	70.00	0.464			
PCT ml/l	PRP	0.31	0.06	0.29	0.06	0.29	0.08	0.559			
MPV fl	PRP	8.55	0.50	8.55	0.40	8.75	0.30	0.663			
PDW fl	PRP	14.50	0.20	14.60	0.25	14.60	0.30	0.588			

Legend: MPV, platelet volume; PCT, plateletcrit; *VEGFA*, vascular endothelial growth factor A; PDW, platelet distribution width; PLT, platelets; QD, Quartile Deviation; WBC, white blood cells; RBC, red blood cells; EOS, eosinophils.

Table S8. Whole blood (WB) and platelet-rich plasma (PRP) parameters values in individuals with particular variants of the *VEGFA* gene polymorphisms in recessive/dominant model.

Parameter	Source	rs699947				*P*. Mann-Whitney U test
		CC		AC+AA		
		Median	±QD	Median	±QD	
PLT 10^9/l	WB	264.00	28.00	228.00	37.00	0.099
PCT ml/l	WB	2.35	0.28	2.22	0.38	0.312
MPV fl	WB	8.90	0.55	9.40	0.75	0.080
PDW fl	WB	16.00	0.15	16.10	0.15	0.861
WBC 10^9/l	WB	6.80	1.15	6.17	1.11	0.099
RBC 10^{12}/l	WB	4.54	0.27	4.69	0.31	0.129
EOS%	WB	2.10	0.70	2.30	1.00	0.279
EOS# 10*9/L	WB	0.14	0.06	0.14	0.06	0.898
PLT 10^9/l	PRP	351.50	54.50	338.50	74.00	0.428
PCT ml/l	PRP	0.31	0.05	0.29	0.07	0.413
MPV fl	PRP	8.70	0.40	8.55	0.45	0.962
PDW fl	PRP	14.60	0.25	14.60	0.25	0.701

Parameter	Source	rs699947				*P*. Mann-Whitney U test
		AA		AC+CC		
		Median	±QD	Median	±QD	
PLT 10^9/l	WB	221.50	38.00	251.50	32.75	0.007
PCT ml/l	WB	2.04	0.31	2.33	0.36	0.037
MPV fl	WB	9.70	0.70	9.00	0.60	0.022
PDW fl	WB	16.15	0.25	16.05	0.15	0.416
WBC 10^9/l	WB	5.38	1.01	6.59	1.13	0.020
RBC 10^{12}/l	WB	4.73	0.41	4.66	0.28	0.340
EOS%	WB	2.60	1.00	2.10	1.05	0.135
EOS# 10*9/L	WB	0.14	0.04	0.14	0.07	0.798
PLT 10^9/l	PRP	349.00	84.00	341.00	54.00	0.540
PCT ml/l	PRP	0.31	0.07	0.29	0.06	0.308
MPV fl	PRP	8.80	0.55	8.50	0.40	0.011
PDW fl	PRP	14.70	0.25	14.50	0.20	0.054

Parameter	Source	rs2010963				*P*. Mann-Whitney U test
		GG		CG+CC		
		Median	±QD	Median	±QD	
PLT 10^9/l	WB	227.00	38.25	257.00	32.00	0.077
PCT ml/l	WB	2.15	0.37	2.34	0.34	0.345
MPV fl	WB	9.65	0.67	8.90	0.52	0.008
PDW fl	WB	16.10	0.15	16.00	0.15	0.824
WBC 10^9/l	WB	6.07	1.17	6.70	1.19	0.368
RBC 10^{12}/l	WB	4.68	0.29	4.66	0.28	0.597
EOS%	WB	2.60	1.28	2.05	0.95	0.043
EOS# 10*9/L	WB	0.16	0.07	0.13	0.06	0.226
PLT 10^9/l	PRP	345.00	77.50	342.50	54.25	0.959
PCT ml/l	PRP	0.30	0.06	0.31	0.05	0.934
MPV fl	PRP	8.60	0.50	8.45	0.45	0.071
PDW fl	PRP	14.60	0.20	14.50	0.20	0.169

Parameter	Source	rs2010963				*P.* Mann-Whitney U test
		CC		CG+GG		
		**Median**	**±QD**	**Median**	**±QD**	
PLT 10^9/l	WB	266.00	22.75	234.00	40.50	0.414
PCT ml/l	WB	2.39	0.34	2.30	0.38	0.967
MPV fl	WB	8.75	0.42	9.25	0.75	0.082
PDW fl	WB	16.00	0.07	16.10	0.15	0.167
WBC 10^9/l	WB	8.03	2.71	6.21	1.11	0.008
RBC 10^{12}/l	WB	4.72	0.13	4.67	0.31	0.608
EOS%	WB	2.10	0.58	2.30	1.13	0.357
EOS# 10*9/L	WB	0.17	0.04	0.13	0.06	0.330
PLT 10^9/l	PRP	375.00	51.00	340.50	66.50	0.730
PCT ml/l	PRP	0.31	0.05	0.30	0.06	0.975
MPV fl	PRP	8.30	0.40	8.60	0.45	0.122
PDW fl	PRP	14.45	0.25	14.60	0.23	0.779

Parameter	Source	rs1413711				*P.* Mann-Whitney U test
		TT		CT+CC		
		**Median**	**±QD**	**Median**	**±QD**	
PLT 10^9/l	WB	220.00	37.00	253.00	32.00	0.003
PCT ml/l	WB	2.03	0.31	2.33	0.36	0.030
MPV fl	WB	9.70	0.65	9.00	0.65	0.008
PDW fl	WB	16.20	0.25	16.00	0.15	0.256
WBC 10^9/l	WB	5.29	0.79	6.69	1.13	0.009
RBC 10^{12}/l	WB	4.75	0.41	4.66	0.27	0.286
EOS%	WB	2.60	1.00	2.10	1.05	0.169
EOS# 10*9/L	WB	0.13	0.04	0.14	0.07	0.997
PLT 10^9/l	PRP	343.50	83.50	343.00	54.50	0.689
PCT ml/l	PRP	0.30	0.06	0.30	0.06	0.500
MPV fl	PRP	8.80	0.60	8.50	0.40	0.022
PDW fl	PRP	14.75	0.30	14.50	0.20	0.021

Parameter	Source	rs1413711				*P.* Mann-Whitney U test
		CC		CT+TT		
		**Median**	**±QD**	**Median**	**±QD**	
PLT 10^9/l	WB	264.00	28.00	228.00	37.00	0.099
PCT ml/l	WB	2.35	0.28	2.22	0.38	0.312
MPV fl	WB	8.90	0.55	9.40	0.75	0.080
PDW fl	WB	16.00	0.15	16.10	0.15	0.861
WBC 10^9/l	WB	6.80	1.15	6.17	1.11	0.099
RBC 10^{12}/l	WB	4.54	0.27	4.69	0.31	0.129
EOS%	WB	2.10	0.70	2.30	1.00	0.279
EOS# 10*9/L	WB	0.14	0.06	0.14	0.06	0.898
PLT 10^9/l	PRP	351.50	54.50	338.50	74.00	0.428
PCT ml/l	PRP	0.31	0.05	0.29	0.07	0.413
MPV fl	PRP	8.70	0.40	8.55	0.45	0.962
PDW fl	PRP	14.60	0.25	14.60	0.25	0.701

Parameter	Source	rs3024998				P. Mann-Whitney U test
		TT		CT+CC		
		Median	±QD	Median	±QD	
PLT 10^9/l	WB	266.00	22.75	234.00	40.50	0.414
PCT ml/l	WB	2.39	0.34	2.30	0.38	0.967
MPV fl	WB	8.75	0.42	9.25	0.75	0.082
PDW fl	WB	16.00	0.07	16.10	0.15	0.167
WBC 10^9/l	WB	8.03	2.71	6.21	1.11	0.008
RBC 10^{12}/l	WB	4.72	0.13	4.67	0.31	0.608
EOS%	WB	2.10	0.58	2.30	1.13	0.357
EOS# 10*9/L	WB	0.17	0.04	0.13	0.06	0.330
PLT 10^9/l	PRP	375.00	51.00	340.50	66.50	0.730
PCT ml/l	PRP	0.3115	0.05	0.30	0.06	0.975
MPV fl	PRP	8.30	0.40	8.60	0.45	0.122
PDW fl	PRP	14.45	0.25	14.60	0.23	0.779

Parameter	Source	rs3024998				P. Mann-Whitney U test
		CC		CT+TT		
		Median	±QD	Median	±QD	
PLT 10^9/l	WB	227.00	38.00	261.50	32.50	0.016
PCT ml/l	WB	2.10	0.32	2.35	0.37	0.111
MPV fl	WB	9.70	0.65	8.90	0.50	0.003
PDW fl	WB	16.10	0.15	16.00	0.20	0.397
WBC 10^9/l	WB	5.98	1.25	6.53	1.13	0.362
RBC 10^{12}/l	WB	4.69	0.30	4.64	0.28	0.255
EOS%	WB	2.60	1.35	1.90	0.90	0.024
EOS# 10*9/L	WB	0.17	0.07	0.13	0.06	0.128
PLT 10^9/l	PRP	349.00	72.50	340.00	54.00	0.668
PCT ml/l	PRP	0.30	0.06	0.31	0.06	0.703
MPV fl	PRP	8.60	0.50	8.40	0.50	0.027
PDW fl	PRP	14.60	0.20	14.50	0.20	0.030

Parameter	Source	rs3025021				P. Mann-Whitney U test
		TT		CT+CC		
		Median	±QD	Median	±QD	
PLT 10^9/l	WB	227.00	9.50	246.00	41.00	0.853
PCT ml/l	WB	2.31	0.16	2.31	0.38	0.907
MPV fl	WB	9.10	0.75	9.10	0.70	0.722
PDW fl	WB	16.00	0.10	16.10	0.15	0.955
WBC 10^9/l	WB	5.13	0.59	6.32	1.15	0.081
RBC 10^{12}/l	WB	4.75	0.21	4.66	0.30	0.589
EOS%	WB	2.00	1.40	2.30	1.00	0.815
EOS# 10*9/L	WB	0.16	0.05	0.14	0.06	0.802
PLT 10^9/l	PRP	344.00	70.00	343.00	67.00	0.288
PCT ml/l	PRP	0.29	0.08	0.30	0.06	0.291
MPV fl	PRP	8.75	0.30	8.55	0.43	0.444
PDW fl	PRP	14.60	0.30	14.60	0.25	0.472

Parameter	Source	rs3025021				*P*. Mann-Whitney U test
		CC		CT+TT		
		Median	±QD	Median	±QD	
PLT 10^9/l	WB	236.50	41.50	240.00	36.75	0.512
PCT ml/l	WB	2.21	0.39	2.32	0.33	0.682
MPV fl	WB	9.05	0.75	9.30	0.80	0.997
PDW fl	WB	16.05	0.20	16.10	0.20	0.354
WBC 10^9/l	WB	6.25	1.57	6.26	1.07	0.777
RBC 10^{12}/l	WB	4.66	0.31	4.71	0.33	0.605
EOS%	WB	1.90	0.65	2.45	1.30	0.102
EOS# 10*9/L	WB	0.12	0.05	0.17	0.08	0.137
PLT 10^9/l	PRP	356.50	74.00	335.00	61.00	0.718
PCT ml/l	PRP	0.31	0.06	0.29	0.05	0.950
MPV fl	PRP	8.55	0.50	8.60	0.40	0.520
PDW fl	PRP	14.50	0.20	14.60	0.25	0.378

Legend: MPV, platelet volume; PCT, plateletcrit; *VEGFA*, vascular endothelial growth factor A; PDW, platelet distribution width; PLT, platelets; QD, Quartile Deviation; WBC, white blood cells; RBC, red blood cells; EOS, eosinophils.

Table S9. The frequencies (%) and medians (± QD) of basic demographic and clinical characteristics of patients in relation to genotypes of the *VEGFA* gene polymorphisms.

rs699947	Parameter	Genotypes						*P* value			
		AA		AC		CC					
		**Median**	**±QD**	**Median**	**±QD**	**Median**	**±QD**	**Kruskal-Wallis test**	**AA vs AC**	**AA vs CC**	**AC vs CC**
	Age years, median ± QD	47.00	5.25	45.00	4.50	46.50	6.50	0.159			
	BMI, median ± QD	25.33	2.97	25.28	1.75	26.96	3.12	0.399			
	Cigarettes n/day, median ± QD	0.00	0.00	0.00	0.00	0.00	0.00	0.783			
	Alcohol units/ week, median ± QD	2.00	1.00	1.00	2.00	0.00	1.00	0.010	1.000	0.024	0.052
		**n**	**%**	**n**	**%**	**n**	**%**	**χ^2 test**			
	Male sex, n (%)	15	51.72	29	72.50	11	32.35	0.298			
	Cigarette smoking, n (%)	6	20.69	11	15.94	5	14.71	0.801			
	Hypertension, n (%)	3	10.34	13	18.84	2	5.88	0.166			
	Physical therapy, n (%)	12	41.38	31	44.93	15	44.12	0.472			
	Manual therapy, n (%)	9	31.03	14	20.29	4	11.76	0.271			
	NSAIDs, n (%)	11	37.93	35	50.72	15	44.12	0.052			
	Vit. B12 suppl., n (%)	4	13.79	19	27.54	5	14.71	0.177			
	Diabetes mellitus, n (%)	0	0.00	1	1.45	3	8.82	0.078			
	Gout, n (%)	3	10.34	0	0.00	5	14.71	0.007			

rs2010963	Parameter	Genotypes						*P* value			
		CC		CG		GG					
		**Median**	**±QD**	**Median**	**±QD**	**Median**	**±QD**	**Kruskal-Wallis test**	**CC vs CG**	**CC vs GG**	**CG vs GG**
	Age years, median ± QD	45.00	3.50	45.00	6.00	47.00	6.00	0.887			
	BMI, median ± QD	26.90	3.83	25.83	1.97	25.19	2.05	0.730			
	Cigarettes n/day, median ± QD	0.00	10.00	0.00	0.00	0.00	0.00	0.008	0.485	1.000	0.217
	Alcohol units/ week, median ± QD	0.00	0.00	0.00	1.50	1.00	2.00	0.007	0.422	0.029	0.125
		**n**	**%**	**n**	**%**	**n**	**%**	**χ^2 test**			
	Male sex, n (%)	6	60.00	21	37.5	28	42.42	0.411			
	Cigarette smoking, n (%)	3	30.00	3	5.36	16	24.24	0.019			
	Hypertension, n (%)	0	0.00	5	8.93	13	19.70	0.096			
	Physical therapy, n (%)	6	60.00	26	46.43	26	39.39	0.905			
	Manual therapy, n (%)	0	0.00	12	21.43	15	22.73	0.783			
	NSAIDs, n (%)	6	60.00	23	41.07	32	48.48	0.202			
	Vit. B12 suppl., n (%)	1	10.00	14	25.00	13	19.70	0.516			
	Diabetes mellitus, n (%)	3	30.00	1	1.79	0	0.00	0.000			
	Gout, n (%)	2	20.00	2	3.57	4	6.06	0.133			

rs1413711	Parameter	Genotypes						P value			
		CC		CT		TT					
		Median	±QD	Median	±QD	Median	±QD	Kruskal-Wallis test	CC vs CT	CC vs TT	CT vs TT
	Age years, median ± QD	46.50	6.50	45.00	4.50	47.00	5.50	0.210			
	BMI, median ± QD	26.96	3.12	25.28	1.82	25.70	2.97	0.339			
	Cigarettes n/day, median ± QD	0.00	0.00	0.00	0.00	0.00	0.00	0.958			
	Alcohol units/ week, median ± QD	0.00	1.00	1.00	2.00	2.00	2.00	0.007	0.069	0.013	0.847
		n	%	n	%	n	%	χ^2 test			
	Male sex, n (%)	11	32.35	28	41.18	16	53.33	0.235			
	Cigarette smoking, n (%)	5	14.71	12	17.65	5	16.67	0.932			
	Hypertension, n (%)	2	5.88	13	19.12	3	10.00	0.149			
	Physical therapy, n (%)	15	44.12	29	42.65	14	46.67	0.385			
	Manual therapy, n (%)	4	11.76	13	19.12	10	33.33	0.224			
	NSAIDs, n (%)	15	44.12	34	50.00	12	40.00	0.064			
	Vit. B12 suppl., n (%)	5	14.71	19	27.94	4	13.33	0.148			
	Diabetes mellitus, n (%)	3	8.82	1	1.47	0	0.00	0.068			
	Gout, n (%)	5	14.74	0	0.00	3	10.00	0.008			

rs3024998	Parameter	Genotypes						P value			
		CC		CT		TT					
		Median	±QD	Median	±QD	Median	±QD	Kruskal-Wallis test	CC vs CT	CC vs TT	CT vs TT
	Age years, median ± QD	46.50	6.25	45.00	5.00	45.00	3.50	0.952			
	BMI, median ± QD	25.33	2.05	25.83	1.97	26.90	3.83	0.763			
	Cigarettes n/day, median ± QD	0.00	0.00	0.00	0.00	0.00	10.00	0.006	0.196	1.000	0.479
	Alcohol units/ week, median ± QD	2.00	2.00	0.00	1.00	0.00	0.00	0.002	0.030	0.019	0.548
		n	%	n	%	n	%	χ^2 test			
	Male sex, n (%)	28	43.08	21	36.84	6	60.00	0.371			
	Cigarette smoking, n (%)	16	24.62	3	5.26	3	30.00	0.008			
	Hypertension, n (%)	13	20.00	5	8.77	0	0.00	0.084			
	Physical therapy, n (%)	25	38.46	27	47.37	6	60.00	0.981			
	Manual therapy, n (%)	16	24.62	11	19.30	0	0.00	0.833			
	NSAIDs, n (%)	32	49.23	23	40.35	6	60.00	0.249			
	Vit. B12 suppl., n (%)	13	20.00	14	24.56	1	10.00	0.551			
	Diabetes mellitus, n (%)	0	0.00	1	1.75	3	30.00	0.000			
	Gout, n (%)	4	6.45	2	3.51	2	20.00	0.131			

rs3025021	Parameter	Genotypes						P value			
		CC		CT		TT					
		Median	±QD	Median	±QD	Median	±QD	Kruskal-Wallis test	CC vs CT	CC vs TT	CT vs TT
	Age years, median ± QD	46.00	5.50	46.00	5.00	43.00	6.00	0.594			
	BMI, median ± QD	25.59	1.82	25.47	2.34	26.53	2.32	0.737			
	Cigarettes n/day, median ± QD	0.00	0.75	0.00	0.00	0.00	0.00	0.078			
	Alcohol units/ week, median ± QD	1.00	1.00	1.00	1.75	0.00	1.00	0.566			
		**n**	**%**	**n**	**%**	**n**	**%**	**χ^2 test**			
	Male sex, n (%)	17	32.69	32	45.71	6	60.00	0.167			
	Cigarette smoking, n (%)	13	25.00	9	12.86	0	0.00	0.070			
	Hypertension, n (%)	10	19.23	7	10.00	1	10.00	0.320			
	Physical therapy, n (%)	26	50.00	30	42.86	2	20.00	0.848			
	Manual therapy, n (%)	13	25.00	12	17.14	2	20.00	0.680			
	NSAIDs, n (%)	25	50.00	34	48.57	2	20.00	0.639			
	Vit. B12 suppl., n (%)	12	23.08	14	20.00	2	20.00	0.915			
	Diabetes mellitus, n (%)	0	0.00	4	5.71	0	0.00	0.161			
	Gout, n (%)	0	0.00	7	10.00	1	10.00	0.063			

Legend: BMI, body mass index; NSAIDs, nonsteroidal anti-inflammatory drugs; QD, quartile deviation; Vit., vitamin; suppl., supplementation.
